# Effect of the bulkiness of alkyl ligands on the excited-state dynamics of ZnO nanocrystals†

**DOI:** 10.1039/d3ra05166h

**Published:** 2024-01-17

**Authors:** Yuto Toyota, Masahiko Sagawa, Shohei Yamashita, Yoshinori Okayasu, Yuki Nagai, Yohei Okada, Yoichi Kobayashi

**Affiliations:** a Department of Applied Chemistry, College of Life Sciences, Ritsumeikan University Kusatsu Shiga 525-8577 Japan ykobayas@fc.ritsumei.ac.jp +81-77-561-3915; b Department of Applied Biological Science, Tokyo University of Agriculture and Technology Tokyo 183-8509 Japan yokada@cc.tuat.ac.jp; c Precursory Research for Embryonic Science and Technology (PRESTO), Science and Technology Agency (JST) 4-1-8 Honcho Kawaguchi Saitama 332-0012 Japan

## Abstract

Organic ligands on the surface of nanocrystals (NCs) are extremely important in influencing various physical properties, such as dispersibility, electrical properties, and optical properties. Recent studies have revealed that a slight difference in the molecular structure of aliphatic organic ligands significantly affects the dispersibility of the NCs. On the other hand, the effects of the difference in the molecular structure of ligands on the excited-state dynamics of NCs remain elusive. In this study, we synthesized a series of colloidal ZnO NCs capped with different alkyl phosphonic acids and investigated their photophysical properties using emission decay measurements and transient absorption spectroscopy. The spectral shape and lifetime of the emission originating from the surface oxygen defects of ZnO NCs are almost the same irrespective of the alkyl phosphonic ligands used, indicating that the electronic states of the surface oxygen defects are not affected by the bulkiness of the ligand. On the other hand, the emission quantum yield correlates with the rate of carrier trapping by oxygen defects, suggesting that the rate of carrier trapping reflects the number of oxygen defects. Revealing the detailed relationship between molecular structures of organic ligands and the optical properties of NCs is important for advanced photofunctional superstructures using semiconductor NCs.

## Abstract 2

The effect of molecular structures of alkyl groups of alkyl phosphonic ligands on excited-state dynamics of colloidal ZnO nanocrystals was investigated.

## Graphical abstract

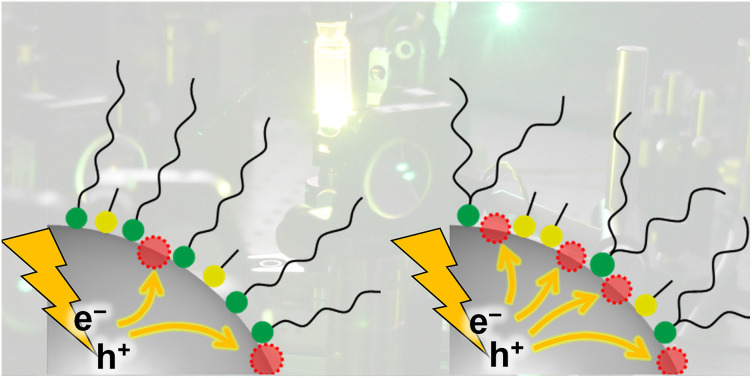

## 1. Introduction

Colloidal semiconductor nanocrystals (NCs) have been extensively studied for decades for many applications, including LEDs, laser media, and solar cells.^1–4^ In recent years, various superstructures of densely-assembled NCs and elegant highly ordered hierarchical mesostructures have been demonstrated.^5,6^ Organic ligands on the surface of NCs play a pivotal role in their dispersibility, optical functions, and electronic properties.^7,8^ Therefore, it is important to clarify the correlation between the molecular structure of the organic ligands and the physical and optical properties of NCs to explore advanced photofunctions of NC mesostructures and to construct nanoarchitectonics^9^ using NCs as building blocks.

Carboxylic acids and phosphonic acids with long alkyl chains have been commonly used as organic ligands to prepare NCs dispersed in organic solvents. These aliphatic organic ligands have been selected empirically depending on the synthesis. Recent detailed studies of organic ligands have revealed that a slight difference in the molecular structure of aliphatic ligands significantly affects the dispersibility of the NCs.^10–13^ Moreover, it has also been reported that the dispersibility and emission properties of NCs are altered by changing the primary, secondary, and tertiary structures of the phosphonic acids and thiols.^14^ However, there are few examples that reveal the effect of the molecular structure of alkyl ligands on the excited-state dynamics of NCs. Revealing the correlation between the molecular structure of organic ligands and photophysical properties is important for the synthesis of NCs and allows arbitrary control of dispersion and photophysical properties.

**Fig. 1:**
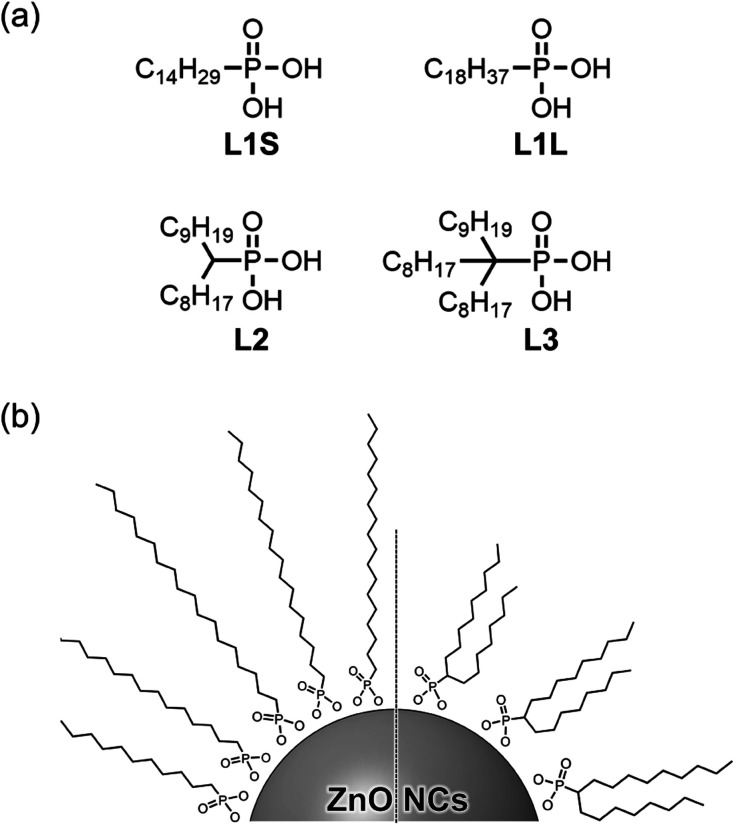
(a) Molecular structures of aliphatic phosphonic acids and (b) schematic image of ZnO NCs coordinated these phosphonic acids.

In this study, we synthesized a series of colloidal ZnO NCs capped with different alkyl phosphonic acids (Fig. 1a) and investigated their photophysical properties in detail using emission decay measurements and transient absorption spectroscopy. Tetradecylphosphonic acid (L1S) and octadecylphosphonic acid (L1L) are primary aliphatic phosphonic acids, and L1S has shorter carbon chains than L1L. Octadecan-9-ylphosphonic acid (L2) is a secondary aliphatic phosphonic acid whose number of carbon atoms is identical to that of L1L. (9-Octyloctadecan-9-yl)phosphonic acid (L3) is tertiary aliphatic phosphonic acid, which is the bulkiest among these ligands. The effect of the alkyl chain length on the optical properties can be investigated by the comparison of the results of L1S-ZnO NCs and L1L-ZnO NCs, while the effect of the bulkiness of the ligands can be investigated by the comparison of the results of L1L-ZnO NCs, L2-ZnO NCs, and L3-ZnO NCs. It is expected that the steric hindrance of alkyl phosphonic acids affects the surface coverage of NCs and the optical properties although long alkyl chains themselves usually do not affect these properties.

## 2. Experimental

### 2.1 Materials

Potassium hydroxide (>85.5%), zinc acetate dihydrate (>99.0%), ethanol (>99.5%), hydrochloric acid (35%), hexane (>95.0%), ethyl acetate (>99.3%), tetrahydrofuran (>99.5%), dichloromethane (>99.5%) and chloroform-*d*_1_ (99.8% D) were purchased from Kanto Chemical Co., Inc. L1S (>98.0%), L1L (>98.0%), 1-bromodecane (>98.0%), triethyl phosphite (>97.0%), diisopropylamine (>99.0%), *n*-buthyllitium (1.59 mol L^−1^ in hexane), 1-iodooctane (>97.0%), bromotrimethylsilane (>95.0%) were purchased from Tokyo Chemical Industry Co., Ltd. All reagents were used without further treatment. The syntheses of L2 and L3 are shown in the ESI.†

### 2.2 Synthesis of ZnO NCs

ZnO NCs were synthesized based on the procedure reported previously.^15^ In a typical synthesis, in a 100 mL flask, potassium hydroxide (0.74 g, 11.5 mmol) was dissolved in 35 mL of ethanol. In a 100 mL flask, zinc acetate dihydrate (1.48 g, 6.7 mmol) was dissolved in 65 mL of ethanol. The potassium hydroxide solution was dropped into the zinc acetate solution quickly and the solution was stirred until a clear solution formed at room temperature. The resulting solution was stirred for 5 hours at 80 °C. The heating and stirring of the solution were stopped and the solution was left for 5 hours to cool down to room temperature. After the supernatant liquid was removed, the suspension was centrifuged once in ethanol (12 000 rcf, 10 min, 20 °C). The precipitate was re-dispersed in methanol and centrifuged once under the same condition and the supernatant liquid was removed.

The precipitate was dispersed in 22 mL of chloroform. The phosphonic acid (0.4 mmol, dissolved in 5 mL of chloroform) was added to the dispersion and stirred for 24 hours at room temperature. 15 mL of acetonitrile was then added to the dispersion, and centrifuged once (12 000 rcf, 10 min, 20 °C). The precipitation was re-dispersed in 5 mL of chloroform, 35 mL of acetonitrile was added, and centrifuged once under the same condition. The precipitation was dried under vacuum overnight at room temperature.

### 2.3 Apparatus

Wide-angle X-ray diffraction (WAXRD) patterns were recorded by an X-ray diffractometer (Rigaku Ultima IV). The size and morphologies of NCs were analyzed by a transmission electron microscope (JEOL, JEM-1400). The concentration of Zn atoms in ZnO NC samples was measured by X-ray fluorescence (XRF) spectroscopy using commercial ZnO as a standard sample to prepare the calibration curve. Organic molecules on the surface of NCs were analyzed by a Fourier transform infrared (FTIR) spectrometer FT/IR-6100 (JASCO). For FTIR measurements, the powder samples were put on the germanium plate (JASCO, PLS-G1) and spectra were obtained using the attenuated total reflection (ATR) method.

Absorption and emission spectra were measured on a UV-3600 spectrophotometer (Shimadzu) and a FP-6500 fluorescence spectrophotometer (JASCO), respectively. For emission measurements, the absorbance at the excitation wavelength was set to 0.08–0.12 using 10 mm quartz cuvettes, and all samples were excited at 350 nm. The emission decays were measured using TSP-2000 (UNISOKU) combined with a 355 nm nanosecond laser (Minilite, Amplitude Japan). The full-width at half maximum (FWHM) of the instrumental response function (IRF) is ∼50 ns. We did not set polarizers before the spectrometer because molecules and NCs can rotate freely on these timescales. The sample was placed in a 10 mm quartz cell, and the absorbance of the solution at the excitation wavelength was set to 1.0. The excitation intensity was 0.1 mJ cm^−2^. Measurements of all solution samples were performed under N_2_ atmosphere.

Electron paramagnetic resonance (EPR) spectrometry was performed on Magnettech ESR5000 (Bruker) at room temperature. The powders of ZnO NCs (5 mg) were placed in a quartz ESR tube (AV-1S, Aguri) and the measurement was conducted under vacuum conditions. The sample was irradiated by UV light emitted from continuous wave 365 nm LED light (CL-1501, Asahi Spectra). The X-band EPR parameters were the magnetic field of 310–370 mT, modulation amplitude of 0.2 mT, modulation frequency of 100 kHz, and microwave power of 0.5 mW.

Transient absorption measurements on femtosecond to nanosecond timescales were conducted by a homemade pump-probe system reported previously.^15^ An amplified femtosecond laser, Spirit One 1040-8 (Spectra-Physics, 1040 nm, the pulse width: ∼270 fs), was split into two beams with a ratio of 1 : 9. The stronger beam was directed to a noncollinear optical parametric amplifier (NOPA), Spirit-NOPA-3H (Spectra-Physics) to generate the 350 nm femtosecond laser pulse for the pump beam. The pump beam was chopped prior to the sample at 500 Hz for signal differencing. The other weaker beam was focused on deuterated water placed in a 10 mm quartz cuvette to generate the white light continuum for the probe beam. Both pump and probe beams were focused on the sample solution placed in a 2 mm quartz cuvette. The polarization between the pump and probe pulses was set at a magic angle. The transmitted probe beam was detected with a multichannel detection system, PK120-C-RK (UNISOKU), composed of a CMOS linear image sensor and a polychromator. The obtained spectra were calibrated for group velocity dispersion using the data obtained by the optical Kerr signal of CHCl_3_ between the pump pulse and the white-light continuum. The instrumental response function was shorter than approximately 100 fs. The sample solutions were stirred with a stirrer during the experiments. The measurements were performed at room temperature.

## 3. Results and discussion

X-ray diffraction (XRD) measurements show that the crystal structures of all ZnO NCs are wurtzite (Fig. S7 in the ESI†). Transmission electron microscopy (TEM) measurements reveal that the shape of ZnO NCs is spherical and the average diameter is *ca.* 7.5 nm (Fig. S6†).

**Fig. 2:**
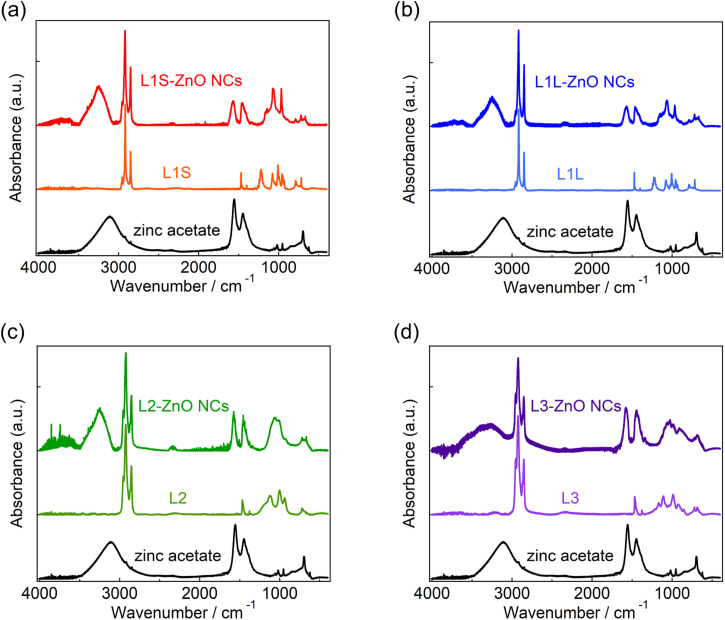
FTIR spectra of (a) L1S-ZnO NCs and L1S, (b) L1L-ZnO NCs and L1L, (c) L2-ZnO NCs and L2, and (d) L3-ZnO NCs and L3 measured by ATR method. FTIR spectrum of zinc acetate, which was used as a zinc precursor, is also shown in all figures.

Fig. 2 shows the ATR-FTIR spectra of zinc acetate, alkyl phosphonic acids (L1S, L1L, L2, and L3), and ZnO NCs capped with these ligands. Following the assignments of the FTIR spectrum of L1S reported previously,^16,17^ the peaks of the FTIR spectra of alkyl phosphonic acids were assigned as follows. In L1S (the middle of Fig. 2a), the sharp peaks at 2800–3000 cm^−1^ are assigned to the C–H stretching vibrational modes of alkyl groups. More specifically, the peaks at 2917 and 2851 cm^−1^ are ascribable to the CH_2_ asymmetric and symmetric stretching modes, and small peaks at 2956 and 2871 cm^−1^ are ascribable to the CH_3_ asymmetric and symmetric stretching modes, respectively. The peak at 1473 cm^−1^ is assigned to the C–H bending mode, the peak at 1225 cm^−1^ is assigned to the P=O stretching mode, and the peaks at 1006 and 956 cm^−1^ are assigned to the P–O–(H) asymmetric and symmetric modes, respectively.

In L1S-ZnO NCs (the top of Fig. 2a), similar peaks ascribable to the C–H stretching modes were observed at 2800–3000 cm^−1^, whereas these peaks were broadened (Fig. S8†). It indicates that the inhomogeneity of the molecular vibrations increased most probably due to the proximity of each ligand by coordination to the surface of NCs. Moreover, the vibrational modes associated with the phosphoryl moiety at 1250–900 cm^−1^ shifted to the lower wavenumber. This result is consistent with the coordination of L1S to the surface Zn atom of ZnO NCs. On the other hand, the relatively broad and intense absorption bands at 1440 and 1580 cm^−1^ cannot be assigned to the L1S. These peaks are rather similar to the symmetric and asymmetric stretching vibrational modes of the carboxylate anion of zinc acetate (the bottom of Fig. 2a). The broad absorption band ascribable to the hydrogen bonding network is also observed at ∼3400 cm^−1^ in L1S-ZnO NCs similar to zinc acetate. Although the absorption bands at 1440 and 1580 cm^−1^ are observed in all ZnO NC samples, the relative amplitudes of these bands respective to those at 2800–3000 cm^−1^ are different. It indicates that the absorption bands at 1400–1600 and 2800–3000 cm^−1^ originate from different species. These results indicate that the peaks at 1440 and 1580 cm^−1^ originate from acetate ions coordinated to the surface of ZnO NCs. Acetate ions originate from the precursor of the zinc (zinc acetate). The FTIR spectra of L1L and L1L-ZnO NCs (top and middle of Fig. 2b) are very similar to those of L1S and L1S-ZnO NCs.

In L2 (the middle of Fig. 2c), the absorption bands at 2800–3000 cm^−1^ associated with the C–H stretching modes are slightly different from those of L1S and L1L due to the different molecular structures (Fig. S8†). The line widths of these peaks are broad even before the coordination to the surface of ZnO NCs most probably because the branched molecular structure itself increases the inhomogeneity of the molecular vibrations. In L2-ZnO NCs (top of Fig. 2c and S8†), the absorption bands associated with the phosphoryl moiety shifted to the lower wavenumbers and the absorption bands associated with acetate ions appeared similar to those of L1S-ZnO NCs and L1L-ZnO NCs. On the other hand, the absorption line widths of the C–H stretching modes of L2-ZnO NCs are almost identical to those of L2.

Similar features were observed in the FTIR spectra of L3 and L3-ZnO NCs, whereas the relative absorption bands at 1400–1600 cm^−1^ respective to those at 2800–3000 cm^−1^ appeared to be larger than those of other ZnO NCs. It suggests that the surface coverage of L3 in L3-ZnO NCs is lower than other ZnO NCs and acetate ions remain on the surface of NCs (*vide infra*). Moreover, the broad absorption band ascribed to the hydrogen bonding network at ∼3250 cm^−1^ is further broadened up to 2500 cm^−1^. Because the broad absorption band at 2500–3100 cm^−1^ of alkyl phosphonic acids was assigned to the vibrational modes of the POH moiety in a previous report,^16^ it suggests that a part of the phosphonic acids of L3-ZnO NCs may be protonated.

The effective abundance ratio of alkyl phosphonic acids and acetate ions in ZnO NC samples was very roughly estimated from the absorption peak area of the CH_2_ symmetric stretching mode (*ν*_CH_, ∼2850 cm^−1^) and that of the asymmetric COO^−^ stretching mode (*ν*_COO_, 1570 cm^−1^) divided by the number of CH_2_ groups per molecule (*N*_CH_, *ν*_CH_,/(*N*_CH_·*ν*_COO_)). *N*_CH_ is 13, 17, 15, and 22 for L1S, L1L, L2, and L3, respectively. Although this value is not proportional to the actual abundance ratio of ligands and acetate ions, it indicates the order of the abundance of ligands per ZnO NC. *ν*_CH_/(*N*_CH_·*ν*_COO_) is 1 (set to the standard), 0.88, 0.88, and 0.36 for L1S, L1L, L2, and L3, respectively. If we simply assume that the surface of ZnO NCs is mainly covered by either phosphonic acid or acetate ions (the OH group was excluded for simplicity), it suggests that the abundance of ligands on the surface of NCs of L3 is much fewer than other samples.

To get more insight into the abundance of ligands on the surface of NCs, surface coverages of ligands were estimated by elemental analyses of Zn atoms in ZnO NCs samples by XRF measurements and quantitative FTIR measurements in deuterated chloroform. The details are shown in the ESI.† Surface coverages of L2 and L3 were estimated to be 4.5 and 1.4 nm^−2^, whereas those of L1 and L2 could not be estimated because of their low solubility. The surface coverage of carboxylate ligands to CdSe NCs is reported to be ∼0.5–4.0 nm^−2^ estimated by proton nuclear magnetic resonance spectroscopy.^18^ Although the obtained values would contain certain uncertainty due to several assumptions, the order of these values appears to be reasonable. Therefore, these results show that the surface coverage of L3 in L3-ZnO NCs is much lower than other samples most probably due to the too-bulky molecular structures.

L2-ZnO NCs were readily dispersed in chloroform, whereas L1S-ZnO NCs and L1L-ZnO NCs took longer times for dispersion. Higher solubility of branched aliphatic ligands is consistent with the previous study.^19^ While L3-ZnO NCs were expected to be well dispersed in solution due to the bulkiest alkyl chains, their dispersibility was similar to those of L1L-ZnO NCs probably because the surface coverage of L3 is much lower than those of other ligands.

**Fig. 3:**
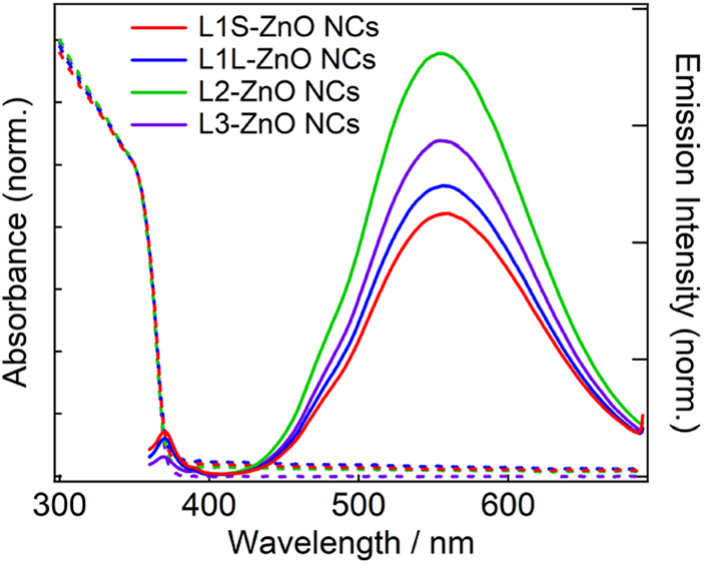
Steady-state absorption and emission spectra of ZnO NCs in chloroform capped with different phosphonic acids at room temperature. The excitation wavelength is 350 nm.

Fig. 3 shows steady-state absorption and emission spectra of ZnO NCs in chloroform. The absorption spectra are exactly the same in different ZnO NC samples. Because the same ZnO NCs were used to prepare different samples, it shows that the ligand exchange reactions did not alter the morphology of ZnO NCs. The bandgap was estimated to be 3.38 eV by Tauc plots (Fig. S10†), which is almost comparable to the bandgap energy of bulk ZnO (3.4 eV).^20^ It is simply because the radius of ZnO NCs (3.2–3.3 nm) is larger than the exciton Bohr radius of ZnO (2.34 nm).

In emission spectra, two emission bands were observed at 370 and 556 nm in all samples. The sharp emission band at 370 nm is ascribed to the excitonic emission because the emission peak did not shift irrespective of the excitation wavelength (Fig. S14†). The broad visible emission band is simply ascribed to the emission originating from the surface oxygen defects. More specifically, several mechanisms are proposed for the origin of the broad emission band of ZnO. van Dijken *et al.* proposed a mechanism that the emission derived from the recombination of an electron at shallow trap states and a hole at deep trap level by showing a very similar relationship between the size dependence of the emission peak and that of the conduction band level.^21^ They also proposed that the hole is firstly trapped by the O^2−^ site on the surface of NCs, sequentially trapped by a pre-existed 
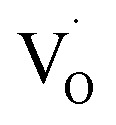
, and then recombines with an electron at the shallow trap level.^21^ It is noted that the Kröger–Vink notation was used for describing the defects.^22,23^ The involvement of the O^2−^ site on the visible emission band is supported by the results of Gamelin *et al.*, where they show that the intensity of the visible emission band of ZnO NCs correlates with the intensity of OH band in the FTIR measurements.^24^ Although the mechanism by van Dijken *et al.*^21^ is convincing, the origin of the deep trap level may be different from the present system because no electron spin resonance (ESR) signals were observed in all samples (both powders and chloroform solutions) at room temperature without light irradiation. (Fig. S11 and S12†). On the other hand, Kamat *et al.* report that the visible emission band originates from the recombination of the electron trapped by the 
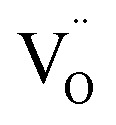
, which corresponds to 
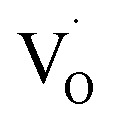
, and the hole at the valence band.^25^ Because it was difficult to determine which mechanisms are dominant, we simply ascribed the broad emission band to the emission originating from the surface oxygen defects.

Similar emission spectra were observed in all ZnO NCs samples. It is consistent with the fact that the difference in the molecular structure of aliphatic moieties of ligands has little influence on the electronic structures of ZnO NCs and defects. On the other hand, the relative emission quantum yields of the defect emission are slightly different in different samples. The relative emission quantum yields were obtained by measuring the emission spectra of freshly prepared solutions three times and average values were described with the standard deviation. The emission spectrum of anthracene in cyclohexane excited at 350 nm was used to obtain the relative emission quantum yield.^26^ The emission quantum yields are 2.9 ± 0.2, 3.2 ± 0.3, 4.7 ± 0.2, and 3.2 ± 0.6% for L1S-ZnO NCs, L1L-ZnO NCs, L2-ZnO NCs, and L3-ZnO NCs, respectively (Table 1). Although the difference is small, reproducible results indicate that these differences originate from the different surface conditions. It is expected that the emission quantum yield of the defect emission increases with the increase in the number of surface oxygen defects. L2 is bulkier than L1S and L1L, and the bulkiness of L2 reduces the density of the surface coverage of ZnO NCs. The reduction of the density of the surface coverage of ligands would increase the number of surface oxygen defects and may increase the quantum yield of the trap emission.

**Table 1:** Time constants of EADS and relative emission quantum yields (*Φ*_EM_) of the visible emission band of ZnO NCs capped with different ligands

Sample	EAS1/ps	EAS2/ps	EAS3/ns	*Φ*_EM_/%
L1S-ZnO NCs	2.1 ± 0.3	62 ± 8	2.4 ± 0.4	2.9 ± 0.2
L1L-ZnO NCs	1.50 ± 0.08	55 ± 10	2.7 ± 0.8	3.2 ± 0.3
L2-ZnO NCs	1.08 ± 0.04	35 ± 3	1.32 ± 0.13	4.7 ± 0.2
L3-ZnO NCs	1.33 ± 0.12	50 ± 7	1.3 ± 0.2	3.2 ± 0.6

On the other hand, the emission quantum yield of L3-ZnO NCs is rather smaller than that of L2-ZnO NCs in spite that L3 has a bulkier structure than L2. FTIR measurements show that a number of acetate ions are coordinated to the surface of L3-ZnO NCs because more acetate ions are coordinated among L3 due to the too-bulky structure of L3. Moreover, the broader absorption band of the hydrogen bonding network suggests that some phosphonic acids may be protonated (POH). The smaller emission quantum yield of the trap emission of L3-ZnO NCs may originate from the acetate ions remaining on the surface of ZnO NCs and the POH moiety, which may somehow decrease the number of oxygen defects. Although the difference in the emission quantum yields is small, they correlate well with the results of time-resolved spectroscopy measurements, which will be discussed later.

**Fig. 4:**
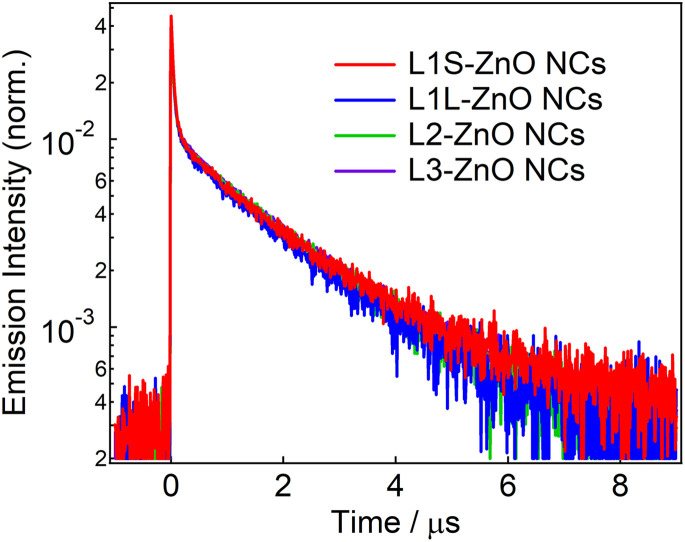
Emission decays of ZnO NCs capped with different ligands in chloroform excited with the 355 nm nanosecond laser pulse (0.1 mJ cm^−2^) and probed at 550 nm.

Fig. 4 shows emission decays of the broad defect emission of ZnO NCs excited at a 355 nm nanosecond laser pulse (0.1 mJ cm^−2^). The probe wavelength is 550 nm. The emission decays are very similar irrespective of the different ligands. The emission decays are fitted with three-exponential decay functions and the obtained lifetimes are summarized in Table S1.† The emission decays on sub-microsecond to microsecond timescales are derived from the relaxation of the deep trap state. The almost identical emission decays of the defect emission in different ligands of ZnO NCs also suggest that the electronic structure of oxygen defects does not depend on the bulkiness of phosphonic ligands. On the other hand, the slightly different relative emission quantum yields suggest that the excited-state dynamics on faster timescales are different in different samples.

**Fig. 5:**
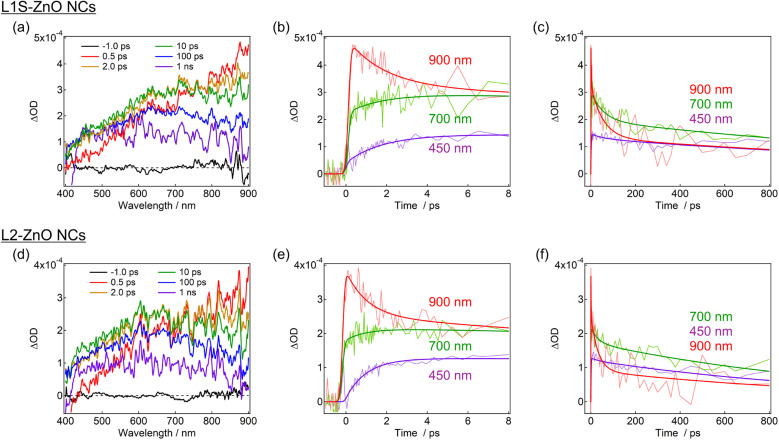
Transient absorption spectra and dynamics of (a–c) L1S-ZnO NCs and (d–f) L2-ZnO NCs in chloroform excited with a 350 nm femtosecond laser pulse (80 nJ per pulse) at room temperature. The bold lines in the transient absorption dynamics are the fitted curves by the global analyses using the three-state sequential decay kinetic model.

Transient absorption measurements were performed to unveil the excited-state dynamics of ZnO NCs faster than nanosecond timescales. As typical examples, the subpicosecond-to-nanosecond transient absorption spectra of L1S-ZnO NCs and L2-ZnO NCs in chloroform excited at 350 nm are shown in Fig. 5. Transient absorption spectra of other samples and detailed analyses are shown in the ESI.†

At 500 fs after the excitation, a broad transient absorption band in visible and near-infrared (NIR) light regions, whose signal gradually increases at the longer wavelength, was observed (Fig. 5a). The transient absorption signal at the NIR light region quickly decays on picosecond timescales and another broad transient absorption with a maximum wavelength at 600–700 nm was observed. The blue shift of the transient absorption band on ultrafast timescales is ascribed to the trapping and localization of carriers generated at the band edge by defects.^27^ After that, the broad transient absorption band longer than 600 nm partly decays on tens of picoseconds and the residual transient absorption remains over nanosecond timescales (Fig. 5b and c). Because the signal derived from the oxygen defects is observed over submicroseconds in the emission decay measurements, the remained transient absorption band is most probably assigned to the trapped carriers by oxygen defects.

The transient absorption spectra of L2-ZnO NCs are very similar to those of L1S-ZnO NCs (Fig. 5c). On the other hand, the transient absorption dynamics of L2-ZnO NCs on picosecond to tens of picosecond timescales are faster than those of L1S-ZnO NCs (Fig. 5d and e).

Global analyses with singular-value decomposition (SVD) were performed using the Glotaran program^28^ to resolve the excited-state dynamics into species-associated spectra. The spectra were tentatively fitted with a three-state sequential decay kinetic model convolved with a Gaussian pulse. The resolved evolution-associated spectra (EAS) are shown in the ESI† and the obtained time constants are summarized in Table 1. The first species (EAS1) is attributed to the excited carriers at the band edge, and the time constant of EAS1 (picoseconds) reflects the carrier trapping by surface defects. The second species (EAS2, tens of picoseconds) has a spectrum intermediate between those of EAS1 and the third species (EAS3, nanoseconds). It should be noted that the global analyses using a simple sequential model of a Glotaran program cannot deal with spectral shifts, line width change, and kinetics in inhomogeneous systems (which shows nonexponential behaviors even in the first-order reaction) rigorously. In the present global analyses, the abovementioned spectral evolutions are tentatively described as additional EAS components. Therefore, it suggests that the carrier trapping by surface defects occurs in a non-exponential manner on the timescales of picoseconds to tens of picoseconds and EAS2 reflects the slower components of the surface trapping. Although the experimental time window was limited to 1 ns in the present study, the EAS3 is most probably ascribed to the surface defects that are observed in emission decay measurements. Similar transient absorption dynamics were observed in L2-ZnO NCs as in L1S-ZnO NCs (Fig. 5d and e). On the other hand, time constants of all EAS of L2-ZnO NCs (1.08 ps, 35 ps, and 1.32 ns) were faster than those of L1S-ZnO NCs (2.1 ps, 62 ps, and 2.4 ns). These results indicate that the surface trapping process of L2-ZnO NCs is faster than that of L1S-ZnO NCs. These results are consistent with the larger emission quantum yield of the defect emission of L2-ZnO NCs than that of L1S-ZnO.

The time constants of EAS and relative emission quantum yields are summarized in Table 1. Interestingly, the time constants of EAS and the emission quantum yields of the defect emission well correlate in all samples, *i.e.*, the larger the emission quantum yields, the smaller the time constants for all EAS. While L3 is the bulkiest in the ligands, the surface coverage of L3 is low, and larger amounts of acetate ions remain on the surface of NCs. Well-correlated time constants of EAS and the relative emission quantum yields clearly indicate that the evolution of the transient absorption spectra reflects the carrier trapping by surface oxygen defects. Moreover, the very similar emission decays in all ZnO NC samples indicate that the nature of the defects is identical and the faster carrier trapping originates from the larger number of surface defects.

While the surface coverage of L3-ZnO NCs is lowest in all samples and a larger number of acetate ions remained on the surface of NCs, the relative emission quantum yield and rate of the surface rapping are intermediate values. It indicates that the coordination by acetate ions and the low surface coverage do not necessarily contribute to the formation of surface oxygen defects. On the other hand, the relative emission quantum yields and transient absorption dynamics are well correlated, while the emission decays are almost identical irrespective of different ligands. These results indicate that molecular structures of the long alkyl moiety of aliphatic phosphonic acids do not affect the electronic states of ZnO NCs and their surface oxygen defects, whereas they alter the number of surface defects.

## 4. Conclusion

**Fig. 6:**
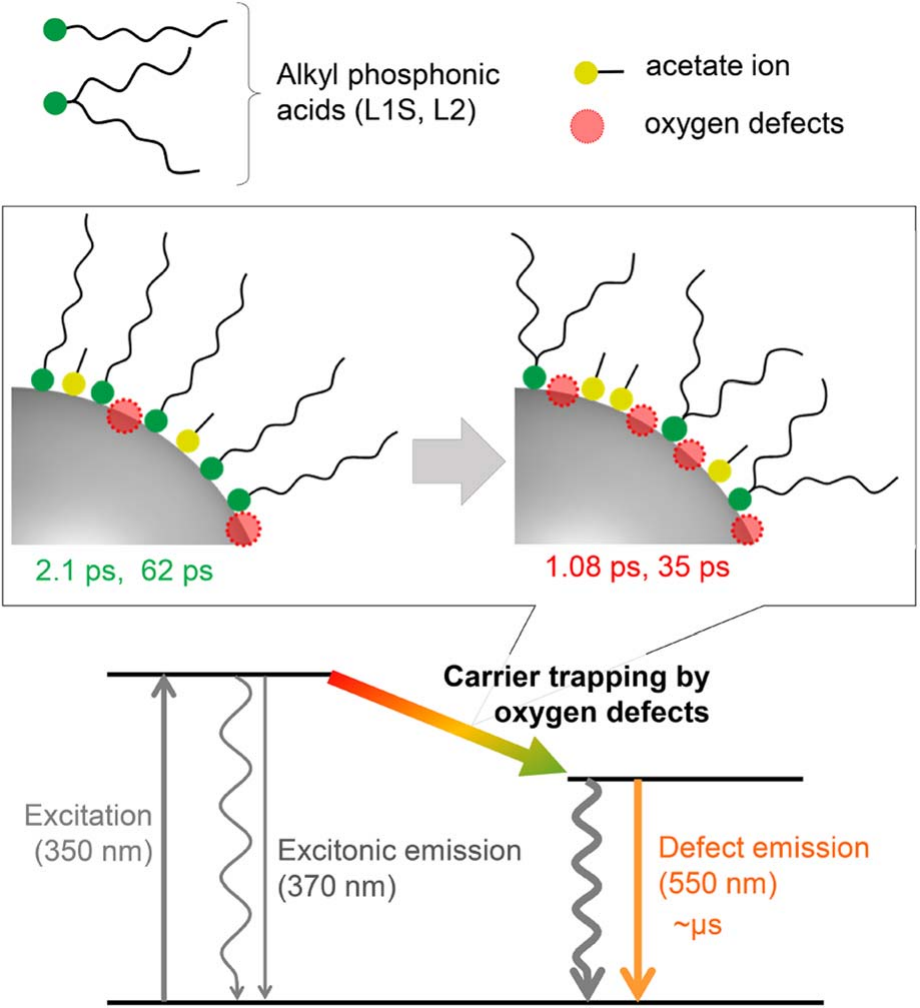
Energy diagram for the excited-state dynamics of ZnO NCs capped with different alkyl phosphonic acids.

A plausible energy diagram of ZnO NCs capped with alkyl phosphonic acid is shown in Fig. 6. When ZnO NCs are excited by UV light, two types of emission bands (exciton and defect emissions) are observed. The emission peak wavelength, width, and lifetime of the defect emission of ZnO NCs are almost the same irrespective of alkyl phosphonic ligands, indicating that the electronic states of the surface oxygen defects are not affected by the bulkiness of the ligand. On the other hand, the emission quantum yield clearly correlates with the rate of carrier trapping by oxygen defects. This correlation is most probably because the number of surface defects changes by the steric effect of aliphatic ligands. Revealing the detailed relationship between molecular structures of organic ligands and the optical properties of NCs is important for advanced hierarchical mesostructures using semiconductor NCs.

## Conflicts of interest

There are no conflicts to declare.

## Supplementary Material

RA-014-D3RA05166H-s001
