# Supplementary material for: Effect of the bulkiness of alkyl ligands on the excited-state dynamics of ZnO nanocrystals
Source: RSC Adv. 2024 Jan 17;14(4):2796–803. doi: 10.1039/d3ra05166h (PMC10792356; doi:10.1039/d3ra05166h)
Supplement: RA-014-D3RA05166H-s001 [file RA-014-D3RA05166H-s001.pdf]

Electronic Supplementary Material (ESI) for RSC Advances.
This journal is © The Royal Society of Chemistry 2024

Supporting Information for:

Effect of the bulkiness of alkyl ligands on excited-state dynamics of ZnO nanocrystals

Yuto Toyota[a], Masahiko Sagawa[b], Shohei Yamashita[b], Yoshinori Okayasu[a], Yuki Nagai[a], Yohei Okada[b,*], Yoichi Kobayashi[a,*]

[a]*Department of Applied Chemistry, College of Life Sciences, Ritsumeikan University, 1-1-1 Nojihigashi, Kusatsu, Shiga 525-8577, Japan.*

[b]*Department of Applied Biological Science Tokyo University of Agriculture and Technology, 3-5-8 Saiwai-cho, Fuchu, Tokyo 183-8509, Japan*

E-mail: ykobayas@fc.ritsumei.ac.jp, yokada@cc.tuat.ac.jp

CONTENTS

1. Syntheses and Characterizations of Phosphonic Acid Ligands **S2**
2. TEM images **S9**
3. XRD Patterns of ZnO NCs **S10**
4. FT-IR Spectra of L2, L3-ZnO NCs in $CDCl_3$ **S11**
5. Estimation of Surface Coverages of Phosphonic Ligands **S12**
6. Tauc Plots of ZnO NCs **S13**
7. Electron Spin Resonance (ESR) Spectra of ZnO NCs **S14**
8. Emission Spectra of ZnO NCs **S15**
9. Emission Decays of ZnO NCs **S16**
10. Femtosecond-to-Nanosecond Transient Absorption Spectra of ZnO NCs **S18**
11. References **S22**

1. Syntheses and Characterizations of Phosphonic Acid Ligands

Bulky ligands L2 and L3 were synthesized based on the procedure reported previously. [1]

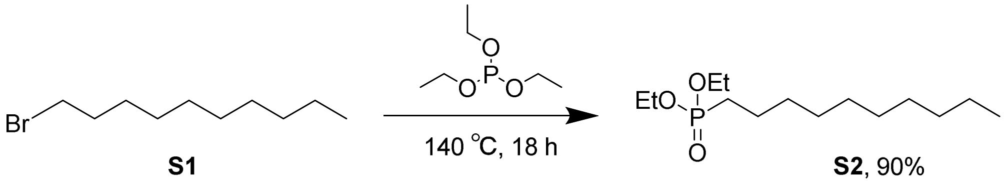

1-Octyldecyl phosphonic acid (L2) was synthesized as follows. 1-Bromodecane (20.0 mmol, 4.13 mL) was dissolved in triethyl phosphite (100 mmol, 17.1 mL) and stirred at 140 °C for 18 h. The reaction was then quenched by the addition of 50 mL of water, followed by three extractions with hexane and brine. The organic layer was dried over Na_2SO_4, filtered, and concentrated in vacuo. Silica gel column chromatography (hexane:EtOAc=2:3, v/v) gave diethyl decylphosphonate as a colorless oil in 90% yield. ^{1}H NMR (500 MHz, $CDCl_3$) δ 4.16–4.03 (4H, m), 1.76–1.67 (2H, m), 1.65–1.54 (2H, m), 1.40–1.21 (20H, m), 0.88 (3H, t, *J* = 7.2 Hz); ^{13}C NMR (125 MHz, $CDCl_3$) δ 61.1, 61.1, 31.7, 30.5, 30.3, 29.3, 29.2, 29.1, 28.9, 26.0, 24.9, 22.4, 22.2, 22.2, 16.3, 16.2, 13.9.

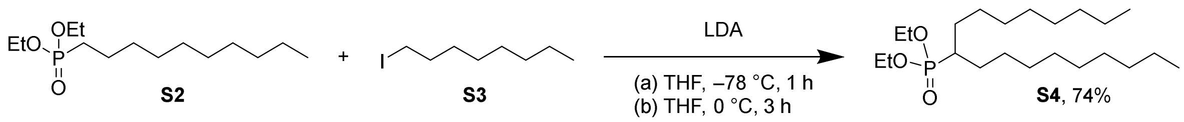

Diisopropylamine (33.1 mmol, 4.65 mL) was dissolved in 20 mL of THF in a flask and stirred at −78 °C. *N*-butyllithium (ca. 15% in hexane) (33.1 mmol, 20.8 mL) was added dropwise, and the mixture was then stirred at 0 °C for 15 min to prepare lithium diisopropylamide (LDA). In a separate flask, 1-iodooctane (24.8 mmol, 4.48 mL) was added to a solution of diethyl decylphosphonate (16.6 mmol, 4.61 g) in THF (100 mL) and stirred at −78 °C. The LDA solution was slowly added dropwise, and the solution was then stirred at −78 °C for 1 h. The mixture was subsequently heated to 0 °C and stirred for 3 h, after which HCl was added to quench the reaction, followed by three extractions with EtOAc and brine. The organic layer was dried over Na_2SO_4, filtered, and concentrated in vacuo. Silica gel column chromatography (hexane:EtOAc=4:1, v/v) gave the resultant diethyl octadecan-9-ylphosphonate as a yellow liquid in 74% yield. ^{1}H NMR (500 MHz, $CDCl_3$) δ 4.15–4.03 (4H, m), 1.74–1.61 (3H, m), 1.53–1.20 (34H, m), 0.88 (6H, t, *J* = 6.5 Hz); ^{13}C NMR (125 MHz, $CDCl_3$) δ 61.3, 61.2, 36.5, 35.4, 31.9, 31.9, 29.7, 29.6, 29.5, 29.4, 29.3, 29.3, 28.2, 28.2, 28.1, 28.1, 27.6, 27.6, 22.7, 16.5, 16.5, 14.1.

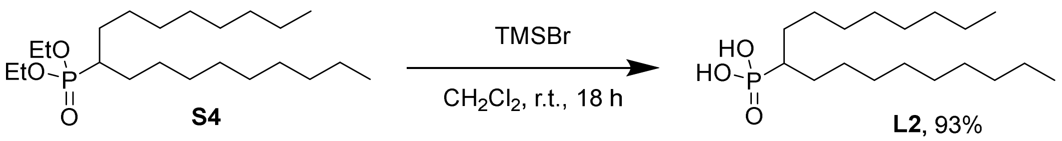

For the third step, TMSBr (37.2 mmol, 4.82 mL) was added to a solution of diethyl octadecan-9-ylphosphonate (12.4 mmol, 4.81 g) in DCM (120 mL) with stirring. After stirring at room temperature for 18 h, 100 mL of water was added to quench the reaction, followed by a single extraction with DCM and brine. The aqueous layer was extracted three more times with EtOAc. The organic layer was dried over Na_2SO_4, filtered, and concentrated in vacuo. Silica gel column chromatography (DCM:MeOH=20:1, v/v) gave the resultant L2 as a yellow liquid in 93% yield. ^{1}H NMR (500 MHz, $CDCl_3$) δ 1.76–1.61 (3H, m), 1.55–1.20 (28H, m), 0.88 (6H, t, *J* = 7.0 Hz); ^{13}C NMR

(125 MHz, $CDCl_3$) δ 36.0, 34.9, 32.1, 30.0, 29.9, 29.8, 29.7, 29.6, 28.1, 27.7, 27.6, 22.9, 14.3.

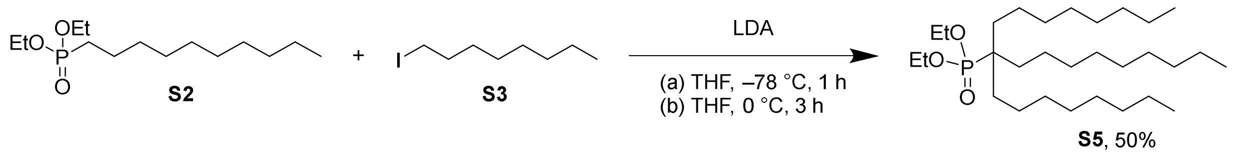

1,1-di(octyl)decylphosphonic acid (L3) was synthesized as follows. Diisopropylamine (59.2 mmol, 8.37 mL) was dissolved in 40 mL of THF in a flask and stirred at −78 °C. N-butyllithium (ca. 15% in hexane) (59.2 mmol, 37.2 mL) was added dropwise, and the mixture was then stirred at 0 °C for 15 min to prepare lithium diisopropylamide (LDA). In a separate flask, 1-iodooctane (4.44 mmol, 8.02 mL) was added to a solution of diethyl decylphosphonate (29.6 mmol, 8.23 g) in THF (120 mL) and stirred at −78 °C. The LDA solution was slowly added dropwise, and the solution was then stirred at −78 °C for 1 h. The mixture was subsequently heated to 0 °C and stirred for 3 h, after which HCl was added to quench the reaction, followed by three extractions with hexane and brine. The organic layer was dried over Na_2SO_4, filtered, and concentrated in vacuo. Silica gel column chromatography (hexane:EtOAc=6:1, v/v) gave the resultant diethyl (9-octyloctadecan-9-yl)phosphonate as a yellow liquid in 50% yield. ^1H NMR (400 MHz, $CDCl_3$) δ 4.12–4.02 (4H, m), 1.55–1.41 (6H, m), 1.40–1.19 (44H, m), 0.88 (9H, t, J = 6.9 Hz) ; ^{13}C NMR (100 MHz, $CDCl_3$) δ 61.2, 61.1, 42.1, 40.7, 34.0, 32.0, 32.0, 30.6, 29.7, 29.6, 29.6, 29.4, 23.8, 23.7, 22.8, 16.7, 16.6, 14.2.

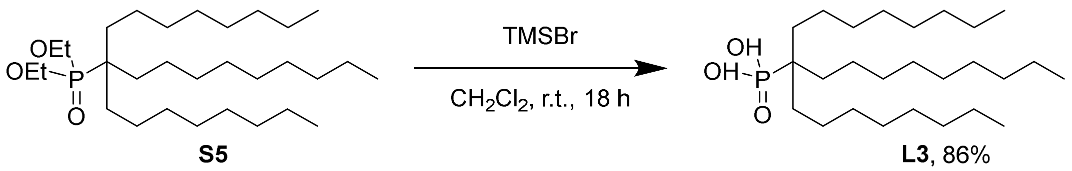

TMSBr (21.5 mmol, 2.78 mL) was added to a solution of diethyl (9-octyloctadecan-9-yl) phosphonate (7.15 mmol, 3.60 g) in DCM (75 mL) with stirring. After stirring at room temperature for overnight, 150 mL of water was added to quench the reaction, followed by twice extractions with DCM/brine. Additionally, the aqueous layer was extracted three times with EtOAc. The organic layer was dried over Na_2SO_4, filtered, and concentrated in vacuo. Silica gel column chromatography (DCM:MeOH=20:1, v/v) gave the resultant L3 as a yellow liquid in 86% yield. ^1H NMR (400 MHz, $CDCl_3$) δ 1.59–1.10 (44H, m), 0.88 (9H, t, J = 6.6 Hz); ^{13}C NMR (100 MHz, $CDCl_3$) δ 34.0, 33.8, 32.5, 32.3, 31.3, 31.2, 31.1, 30.3, 30.0, 29.9, 29.8, 24.3, 24.1, 24.0, 23.0, 14.3.

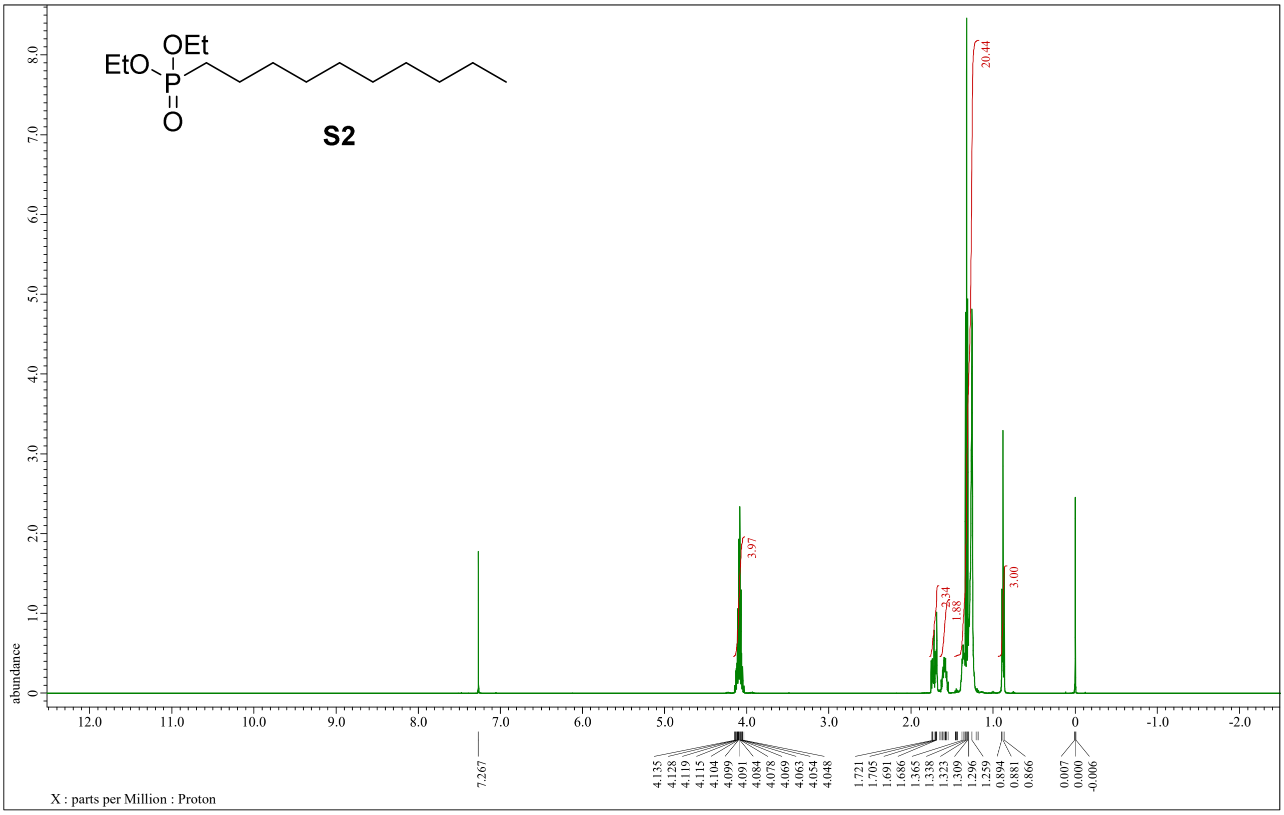

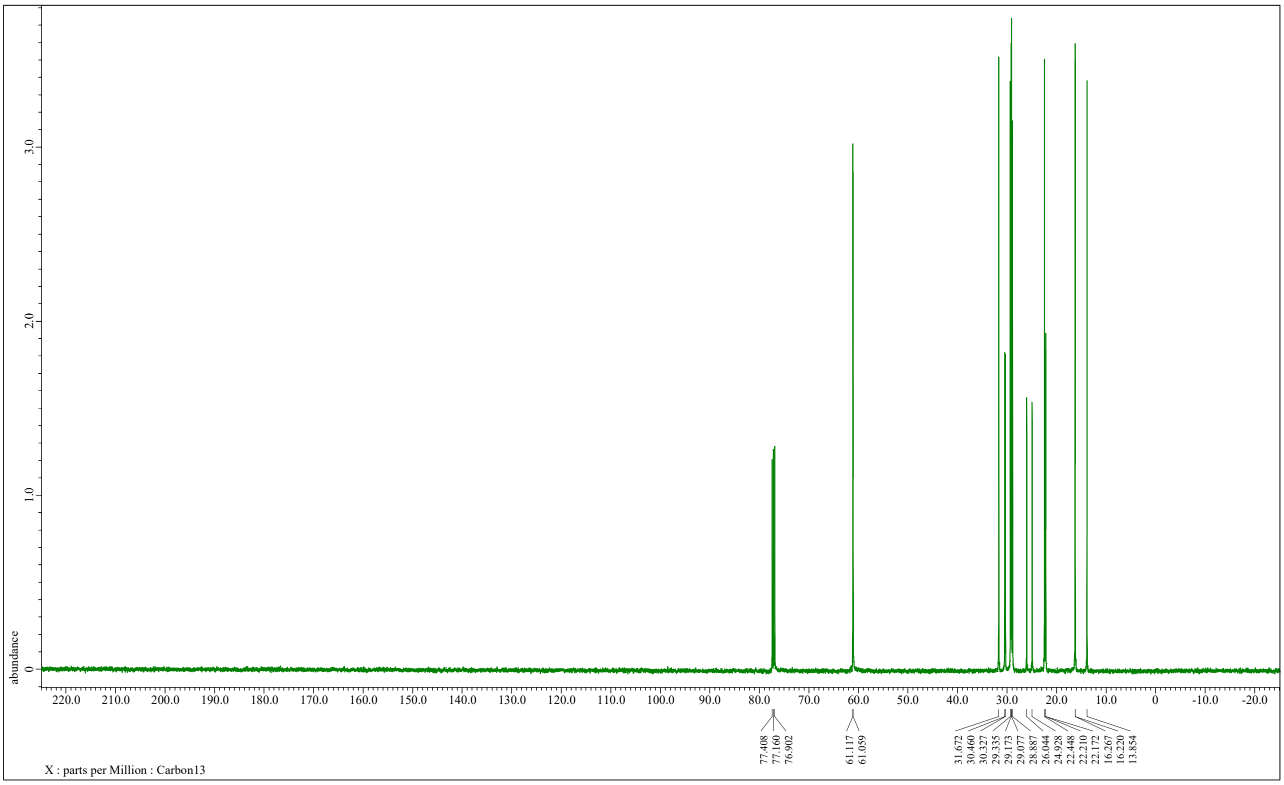

Figure S1. ^{1}H, ^{13}C NMR Spectra of S2

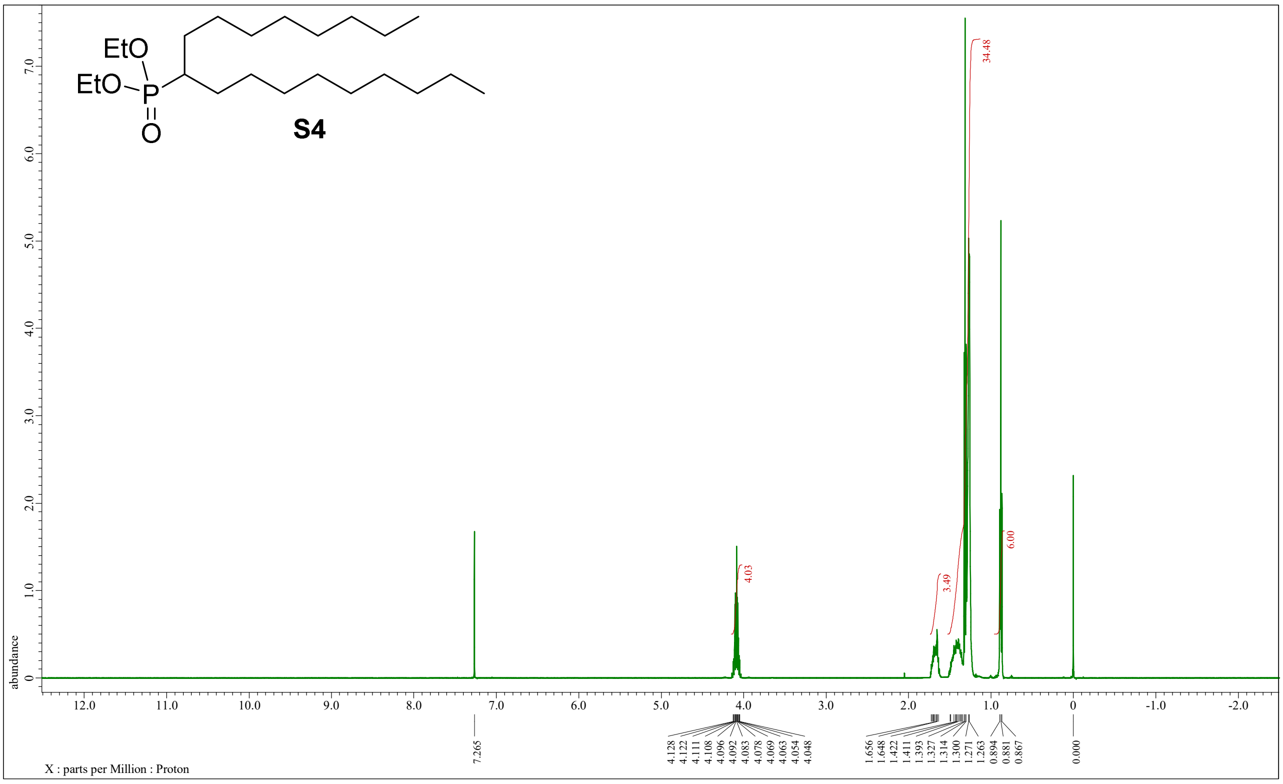

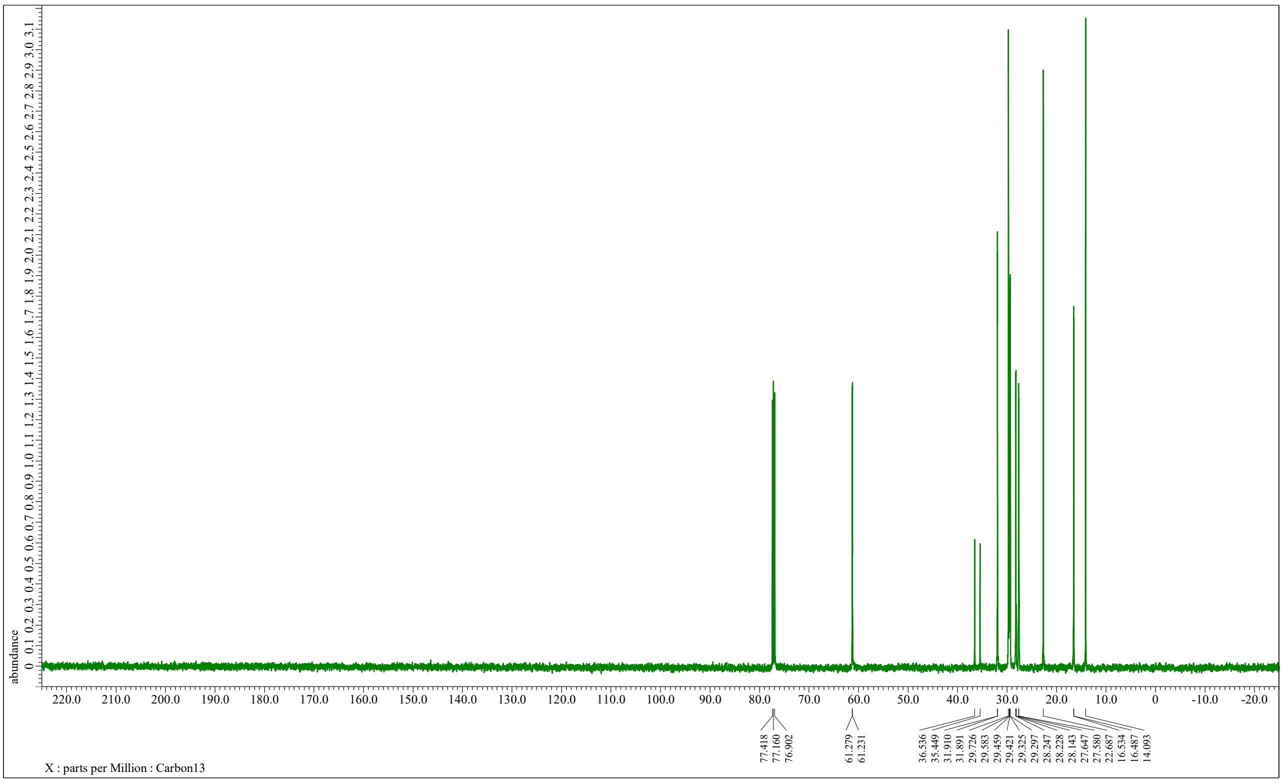

Figure S2. ^{1}H, ^{13}C NMR Spectra of S4

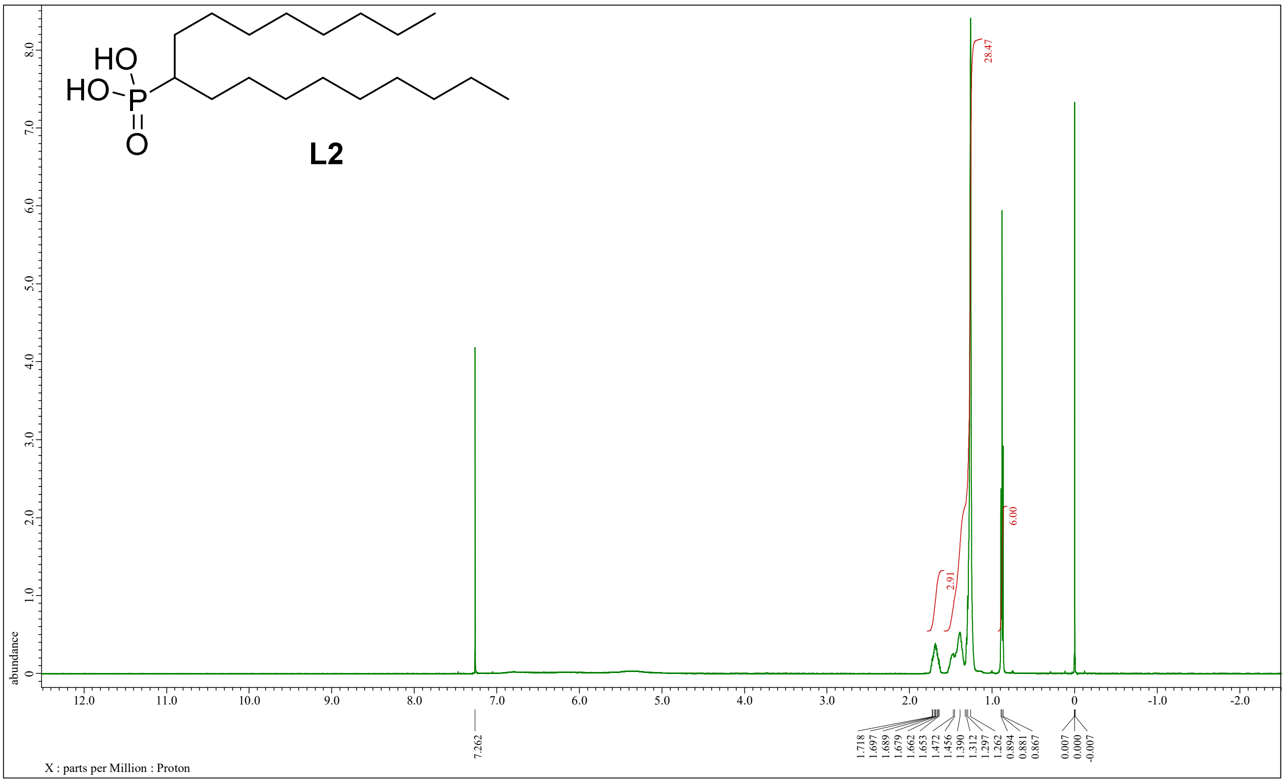

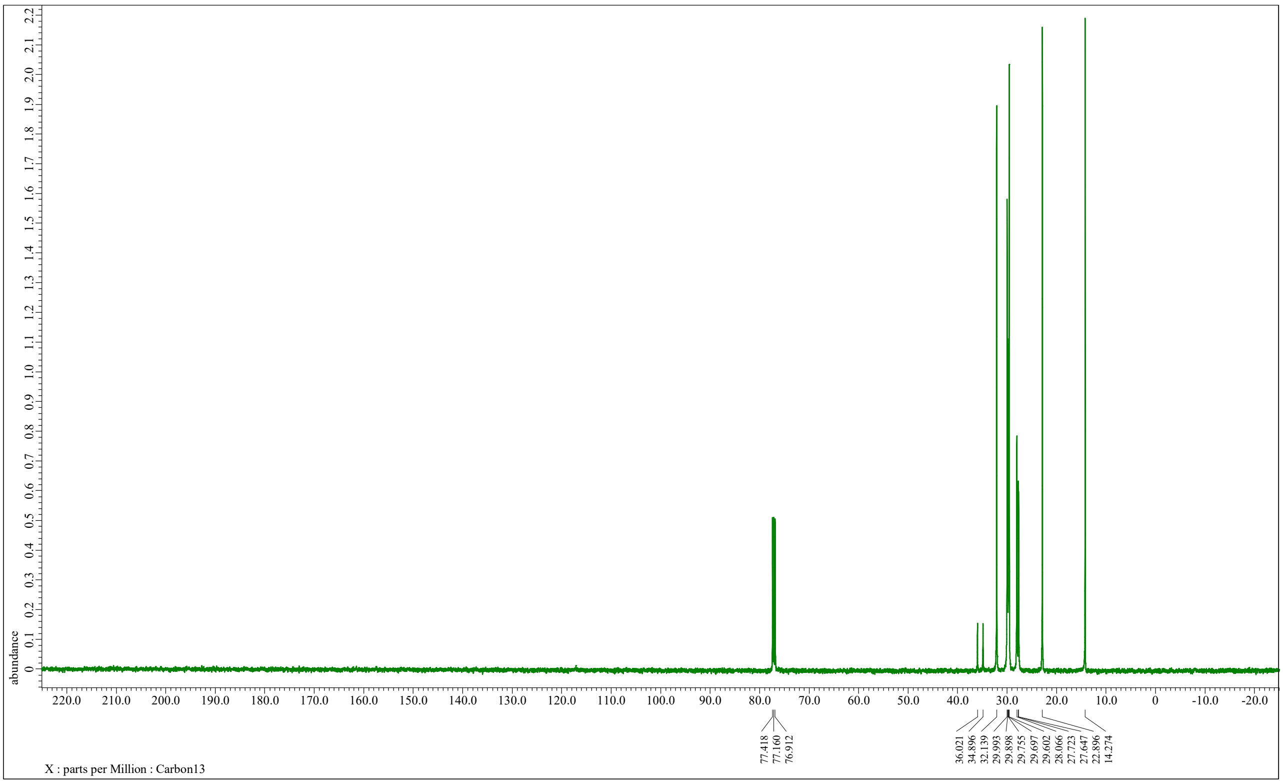

Figure S3. ^{1}H, ^{13}C NMR Spectra of L2

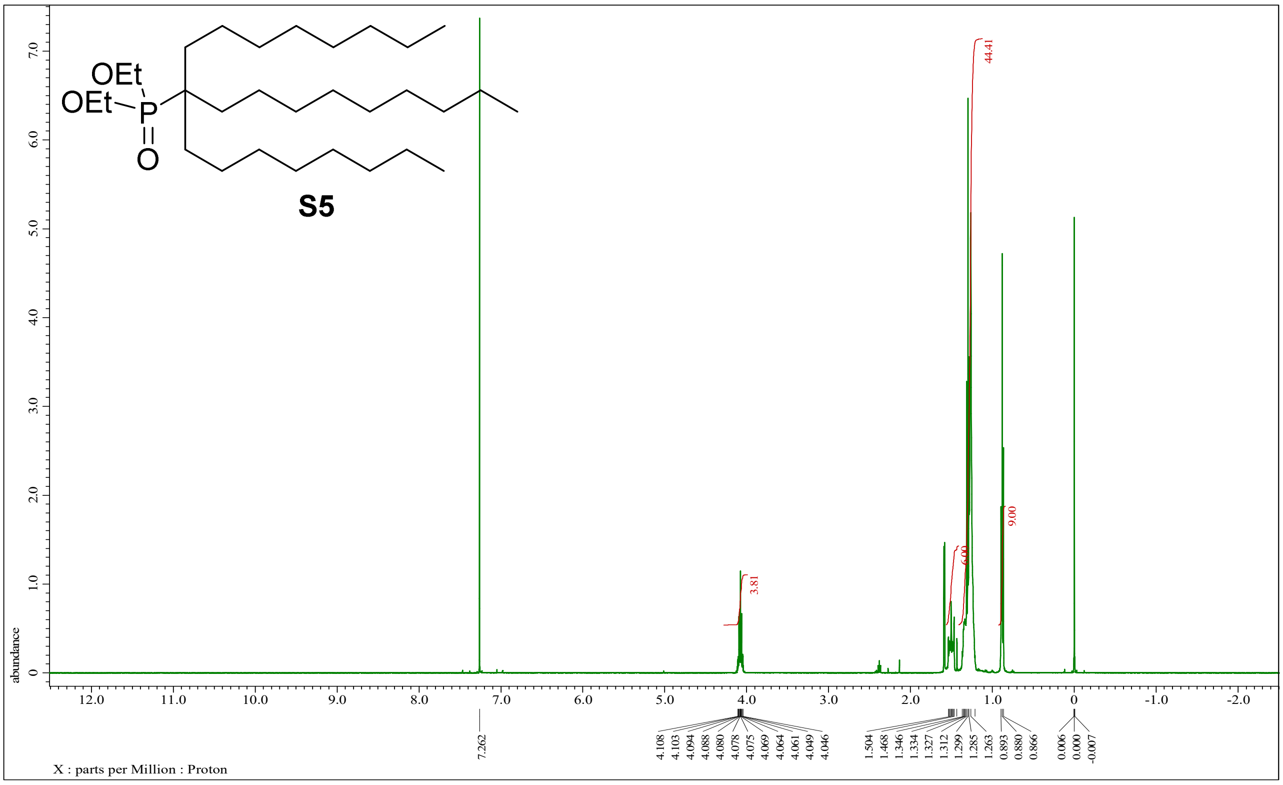

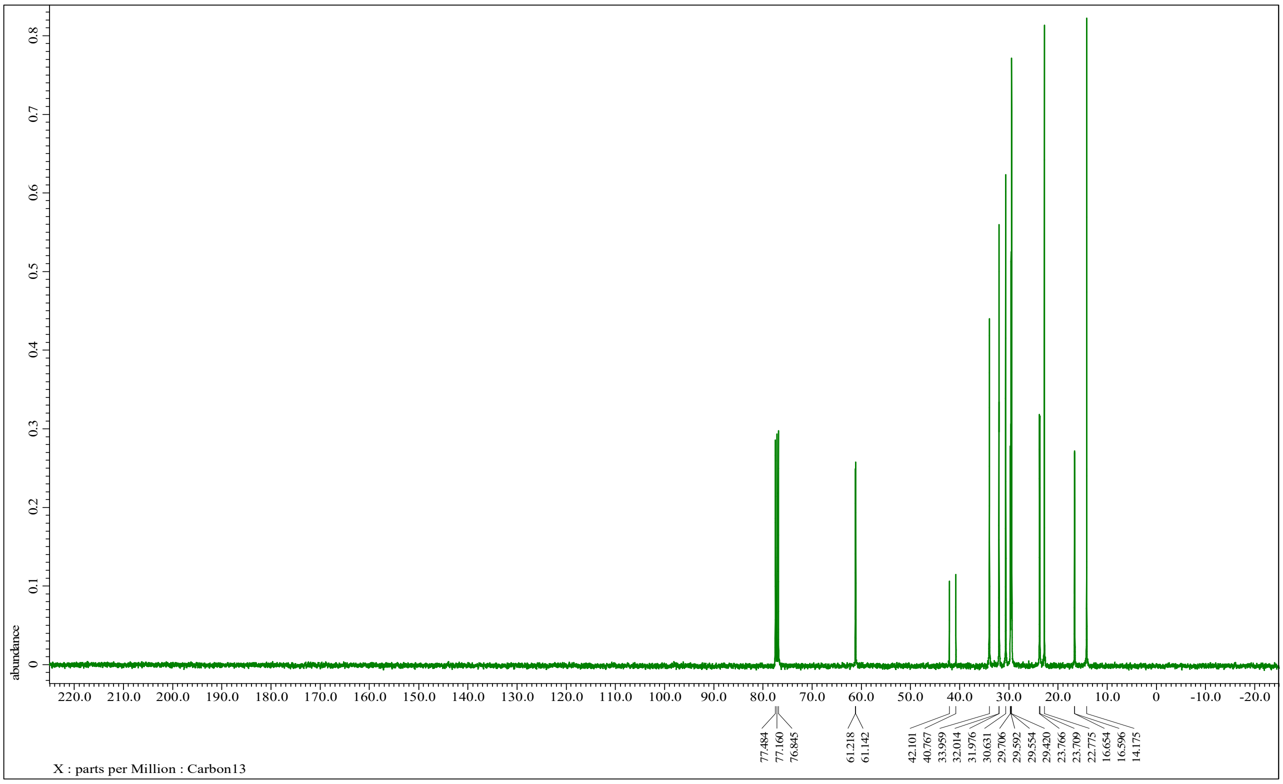

Figure S4. ^{1}H, ^{13}C NMR Spectra of S5

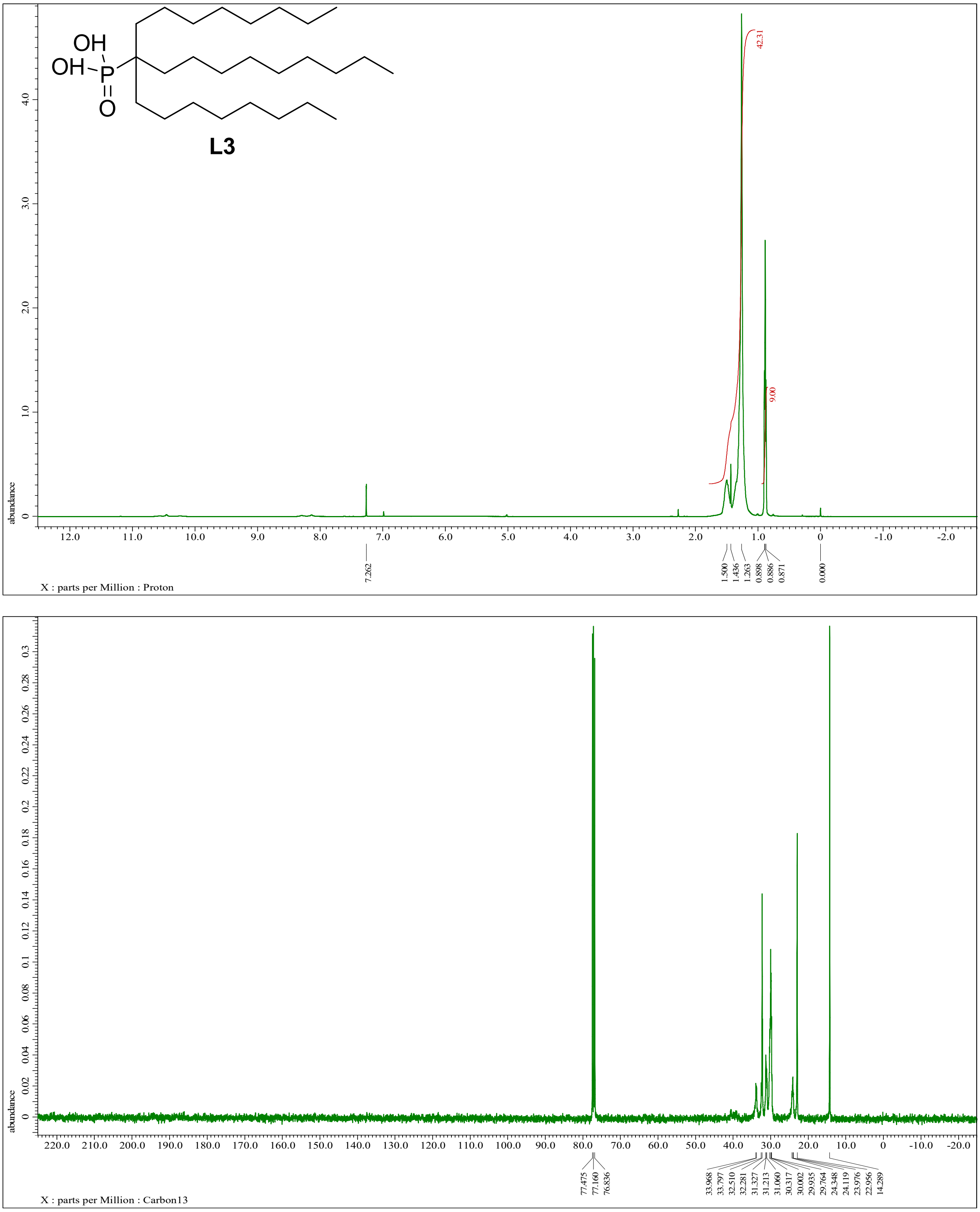

Figure S5. ^{1}H, ^{13}C NMR Spectra of L3

2. TEM images

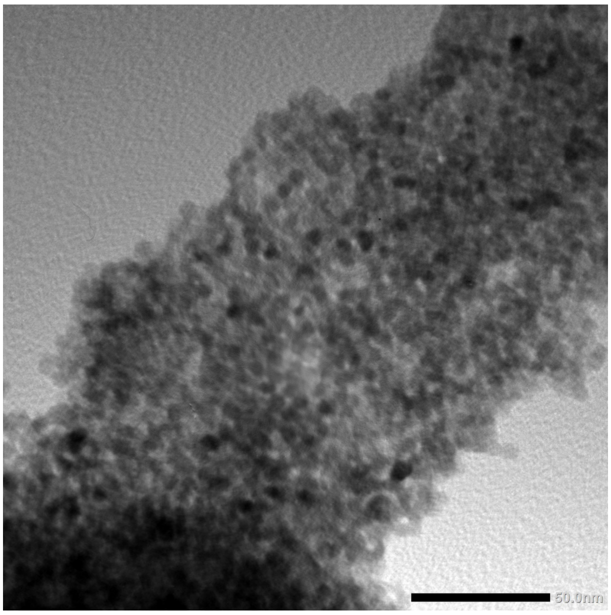

Figure S6 (a) TEM image of ZnO NCs in EtOH

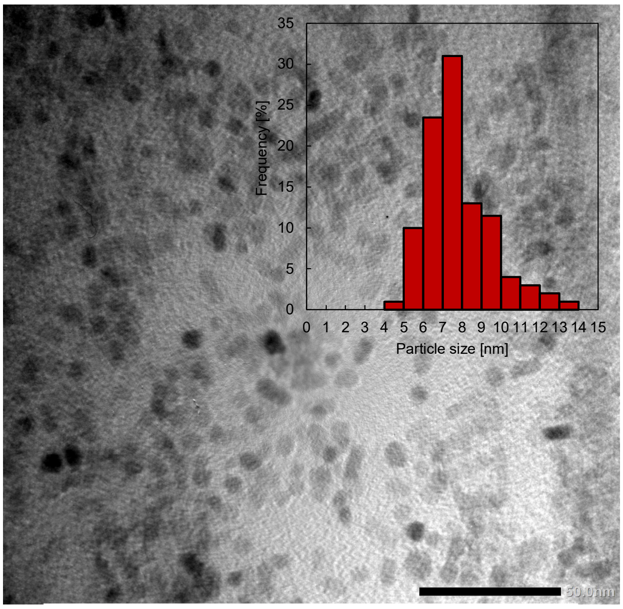

Figure S6 (b) TEM image of L1L-ZnO NCs

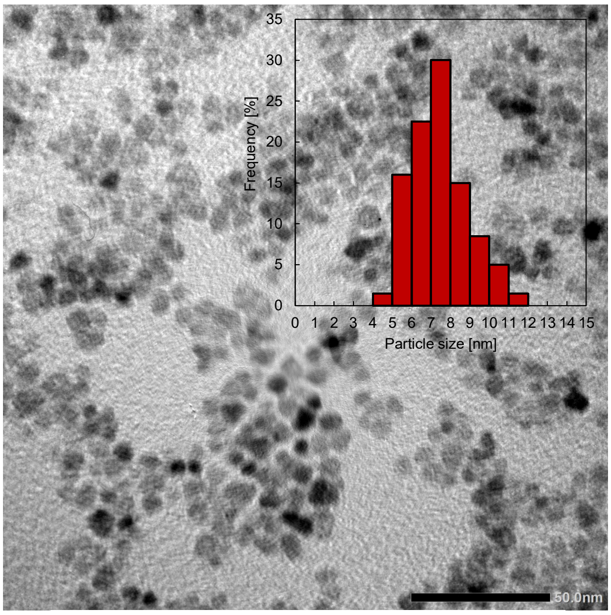

Figure S6 (c) TEM image of L2-ZnO NCs

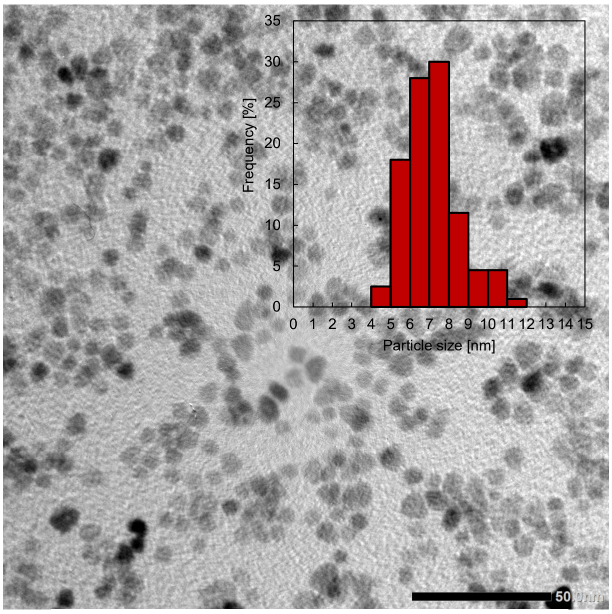

Figure S6 (d) TEM image of L3-ZnO NCs

3. XRD Pattern of ZnO NCs

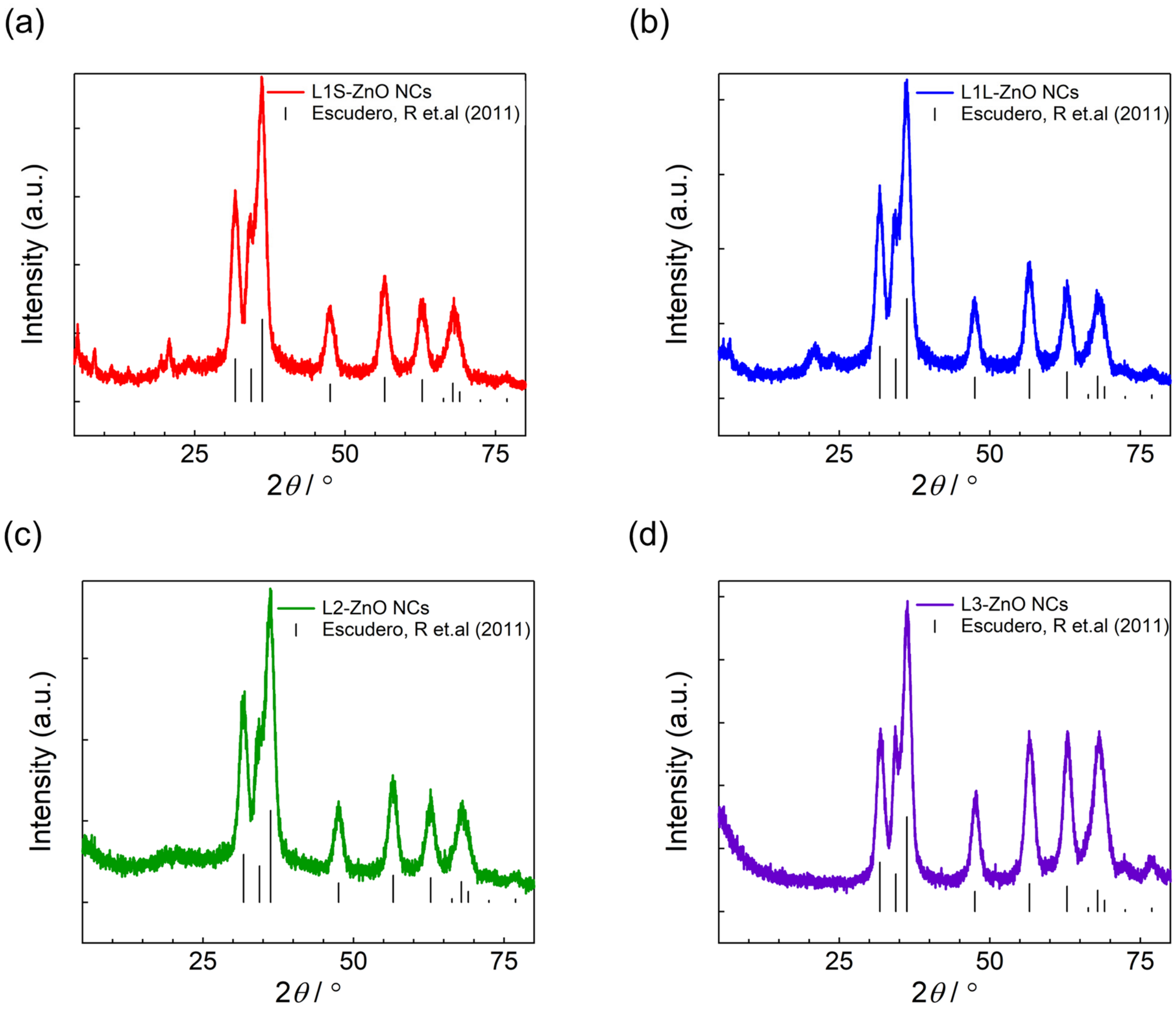

Figure S7. XRD Pattern of L1S–ZnO NCs

4. FT-IR Spectra

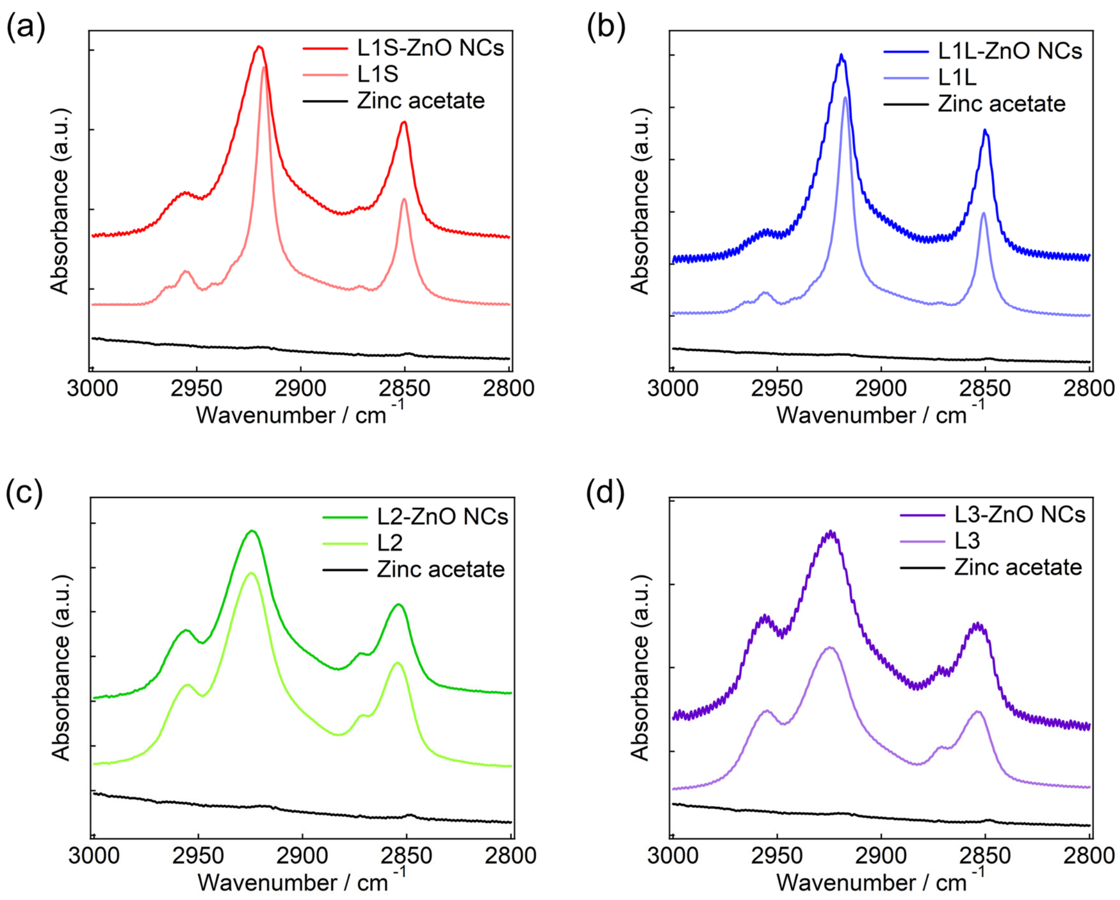

Figure S8. ATR-FTIR Spectra of ligands, ZnO NCs, and zinc acetate at C−H band regions

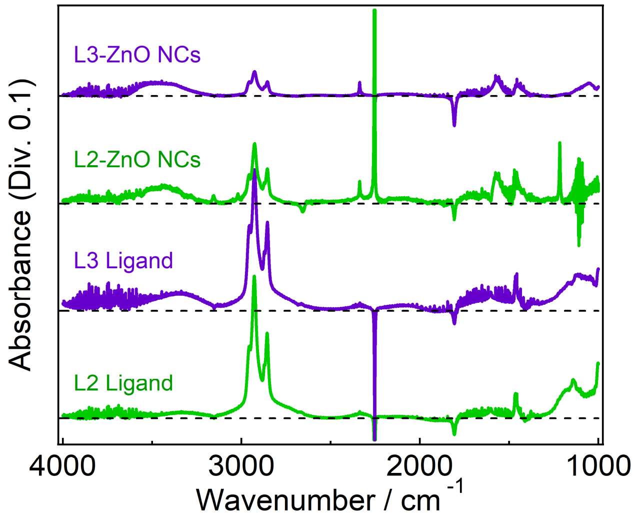

Figure S9. FTIR Spectra of L2, L3, L2-ZnO NCs, and L3- ZnO NCs in $CDCl_3$. For preparation of samples, 3 mg of ligands or ZnO NCs were dissolved in 1 mL of deuterated chloroform. The IR spectra were measured with a 200 μm calcium fluoride liquid cell. (S. T. Japan, 162-1200).

5. Estimation of Surface Coverages of Phosphonic Ligands

By assuming several hypotheses and conducting several additional experiments, we briefly estimated the surface coverages of ligands. The calculations are shown below using the value of L2-ZnO NCs as an example. By assuming that the shape of NCs is spherical, the volume and the surface area of an NC with a diameter of 7.5 nm are estimated to be 221 nm^3 and 177 nm^2, respectively. The molecular weight of a ZnO core without any surface ligands is estimated to be 7.50×10^5.

We conducted the quantitative X-ray fluorescence (XRF) measurements of ZnO NCs powders to estimate the concentration of ZnO by a calibration curve using commercial ZnO. The wt% of ZnO in the L2-ZnO NC powder is 70.1 wt%. It indicates that the number of ZnS NCs per 1-mg L2-ZnO NC powder is 5.62×10^{17}.

We also conducted the quantitative FTIR measurements using a liquid cell with a 200-μm spacer containing 6.37 mM of L2 in deuterated chloroform. The molar absorption coefficient was estimated to be 1.04×10^3 M^{-1} cm^{-1} at 2850 cm^{-1} (CH_2 symmetric stretching mode). Using this value, the concentration of L2 was estimated to be 2.65 mM in the deuterated chloroform containing 3.62 mg of the powder of L2-ZnO NCs in the 1.0-mL solution. If we assume that all L2 is coordinated to the surface of ZnO NCs, the surface coverage is estimated to be 4.46 nm^{-2}. The coverage of oleic acids on the surface of CdSe NCs is reported to be 0.5-4 nm^{-2} estimated by proton nuclear magnetic resonance spectroscopy.[3] Although such a high value may indicate that it was overestimated, the order of this value appears to be reasonable. The surface coverage of L3 in L3-ZnO NCs is much lower than that of L2 in L2-ZnO NCs (1.38 nm^{-2}). This result clearly shows that the surface coverage of L3 in L3-ZnO NCs is much lower than that of L2 in L2-ZnO NCs most probably due to the tertiary carbon ligands. However, the molar absorption coefficients of L1S and L1L could not be obtained because of the low solubility in deuterated chloroform, and therefore, we could not estimate the surface coverages of these ligands.

6. Tauc Plot of L2-ZnO NCs

A bandgap of L2-ZnO NCs was obtained by a Tauc plot. Because ZnO is a direct bandgap semiconductor, the square value of the molar coefficient, α, multiplied by the energy, $(\alpha h\nu)^2$, was plotted as a function of energy. We simply use $(\mathrm{OD}h\nu)^2$ instead of $(\alpha h\nu)^2$ because is α proportional to OD. The bandgap was estimated to be 3.38 eV by the tangent of the edge of the Tauc plot. Because the absorption edges of all samples were the same. The bandgaps of other samples are also 3.38 eV.

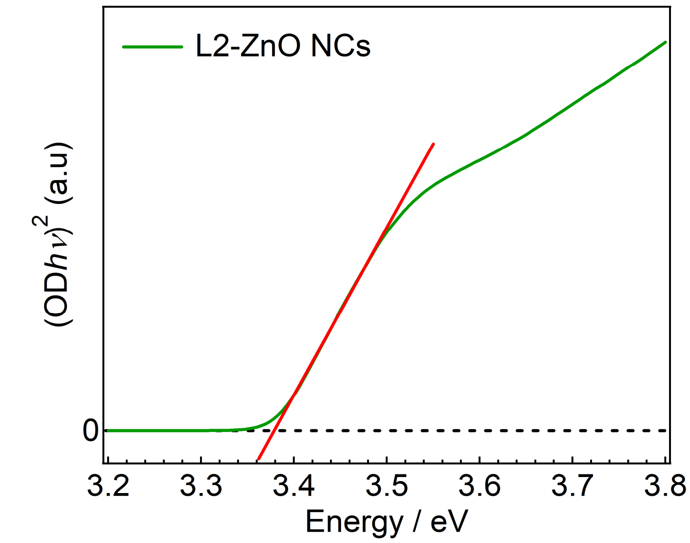

Figure S10. Tauc plot of L2-ZnO NCs

7. Electron Spin Resonance (ESR) Spectra of ZnO NCs

Before the irradiation, no spin signal was observed in both vacuum and chloroform. After 365 nm irradiation, the signal was observed at 1.96 g (345 mT) when the ZnO NCs were in vacuum. However, the signal was not observed in chloroform.

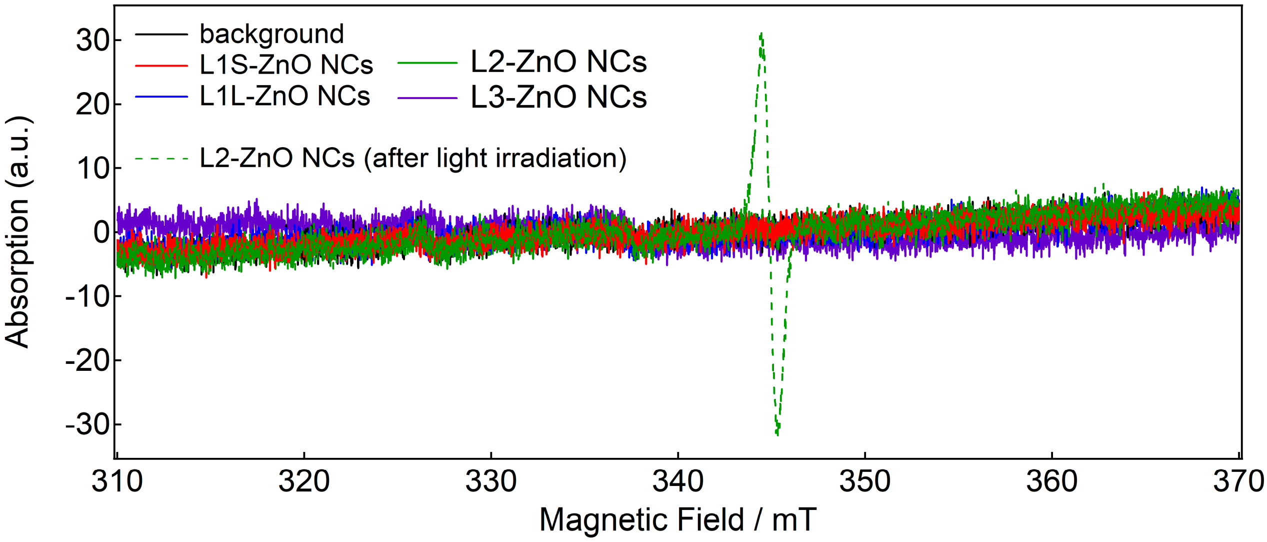

Figure S11. ESR spectra of the powders of ZnO NCs under vacuum before and after irradiation of 365-nm continuous wave (CW) light (2.0 mW) for 60 s.

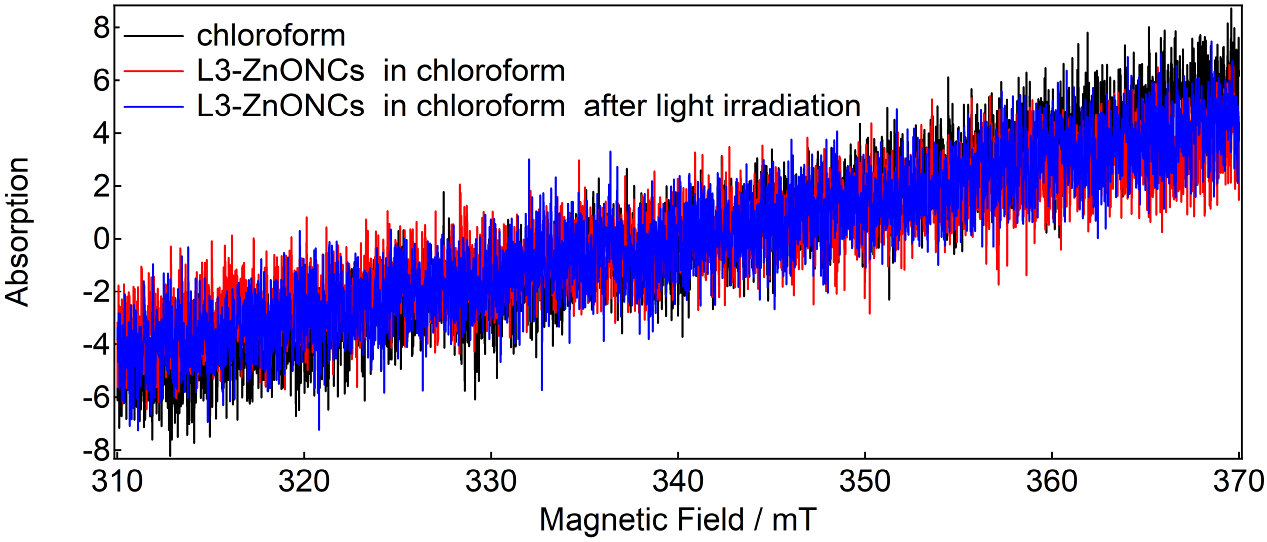

Figure S12. ESR spectra of L3-ZnO NCs in chloroform before and after irradiation of 365-nm continuous wave (CW) light (2.0 mW) for 60 s.

8. Emission Spectra of ZnO NCs

Emission spectra were measured three times by preparing fresh samples each time. The relative emission quantum yields were calculated by the average of these results.

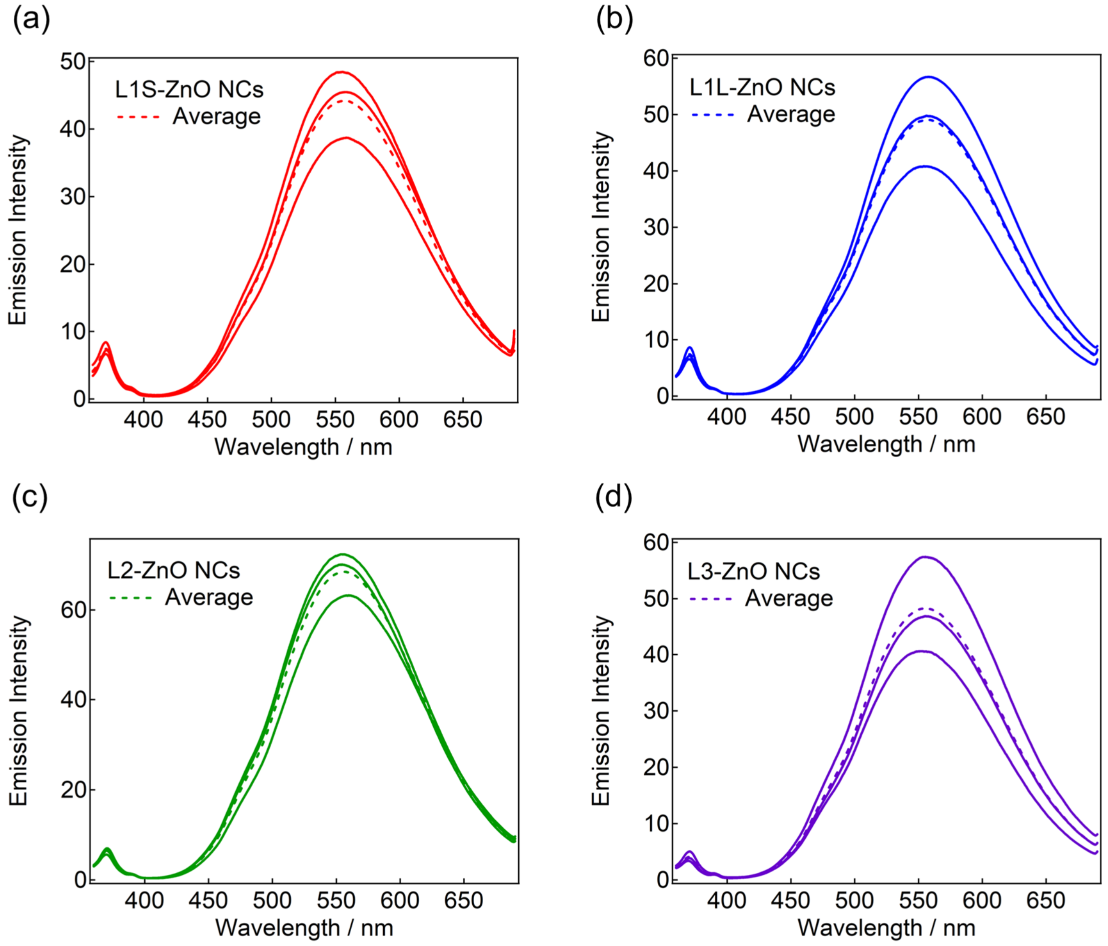

Figure S13. Emission of ZnO NCs in $CDCl_3$ (excited at 350 nm)

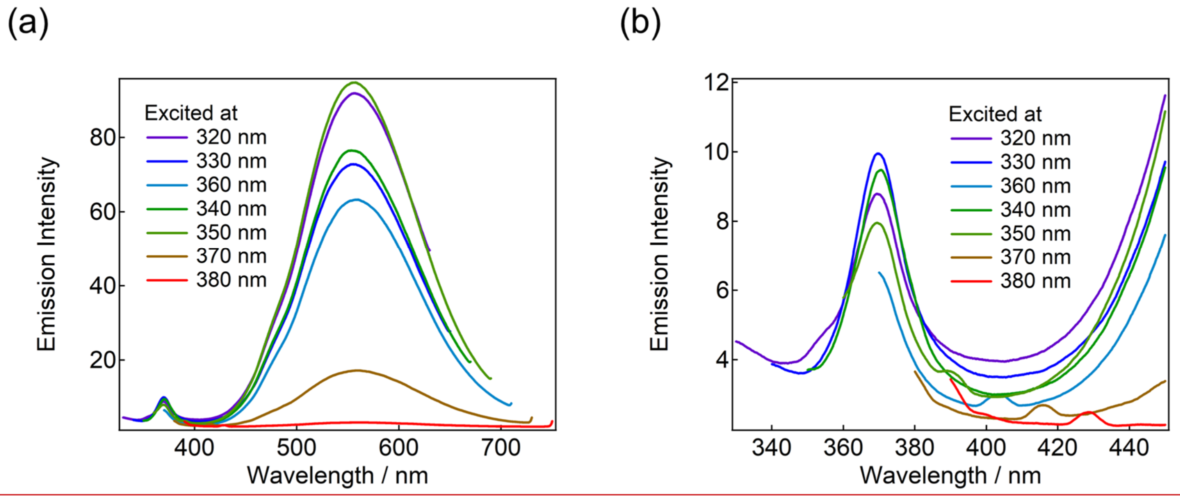

Figure S14. (a) Emission spectra of L2-ZnO NCs excited at different wavelengths. (b) magnified emission spectra at the shorter wavelength region.

9. Emission Decays of ZnO NCs

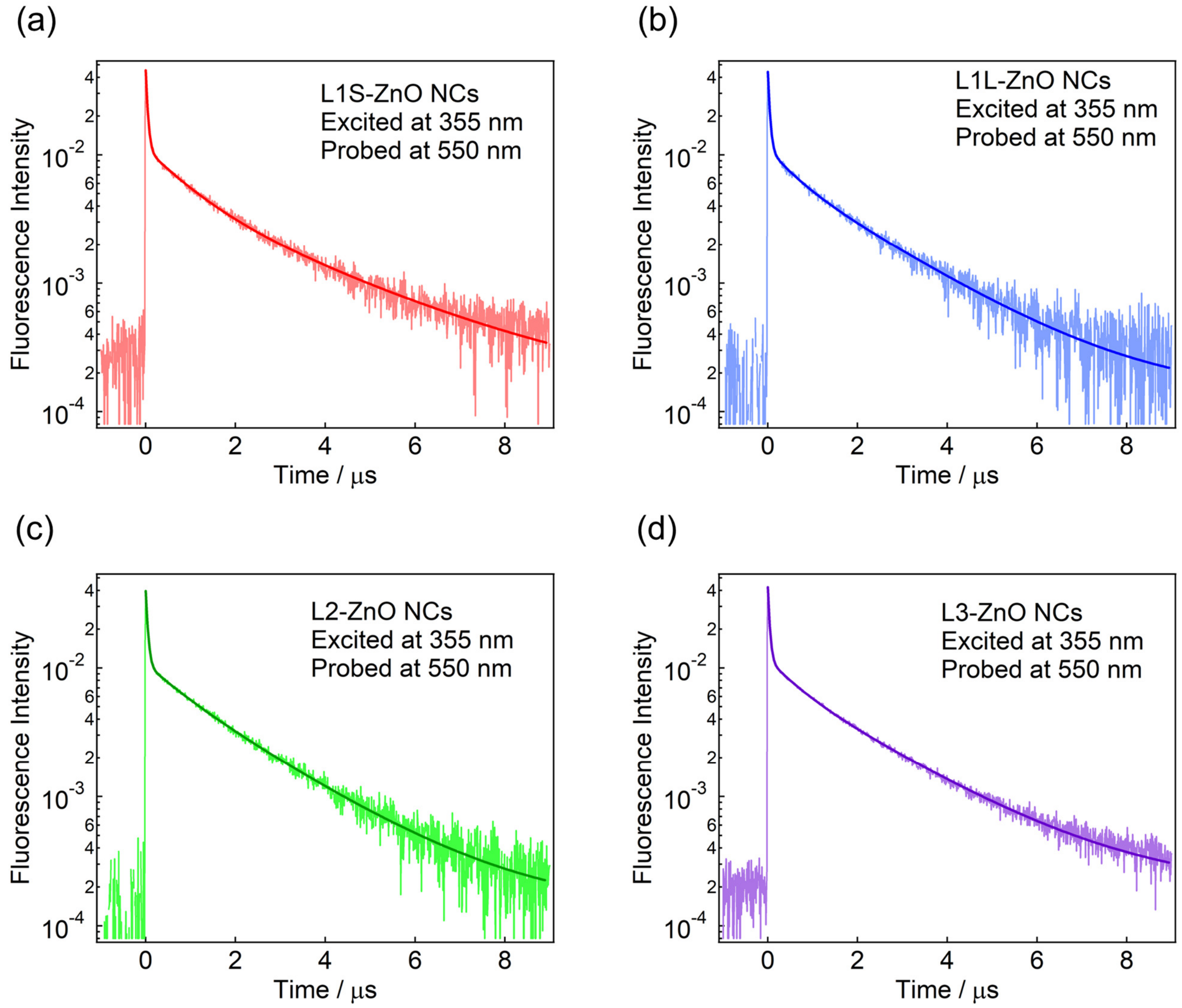

Figure S15. Emission decays of ZnO NCs capped with different alkyl phosphonic acids in chloroform excited with a 355-nm nanosecond laser pulse (0.1 mJ/cm^2). The probe wavelength is 550 nm.

Table S1. Time constants of emission decays of ZnO NCs.

sample	τ_1 / μs	τ_2 / μs	τ_3 / μs
L1S-ZnO NCs	0.040 (73.8%)	0.342 (7.2%)	1.815 (19.0%)
L1L-ZnO NCs	0.041 (74.5%)	0.543 (8.6%)	2.001 (16.8%)
L2-ZnO NCs	0.044 (73.0%)	0.775 (7.1%)	1.995 (19.8%)
L3-ZnO NCs	0.040 (72.7%)	0.626 (8.9%)	2.107 (18.4%)

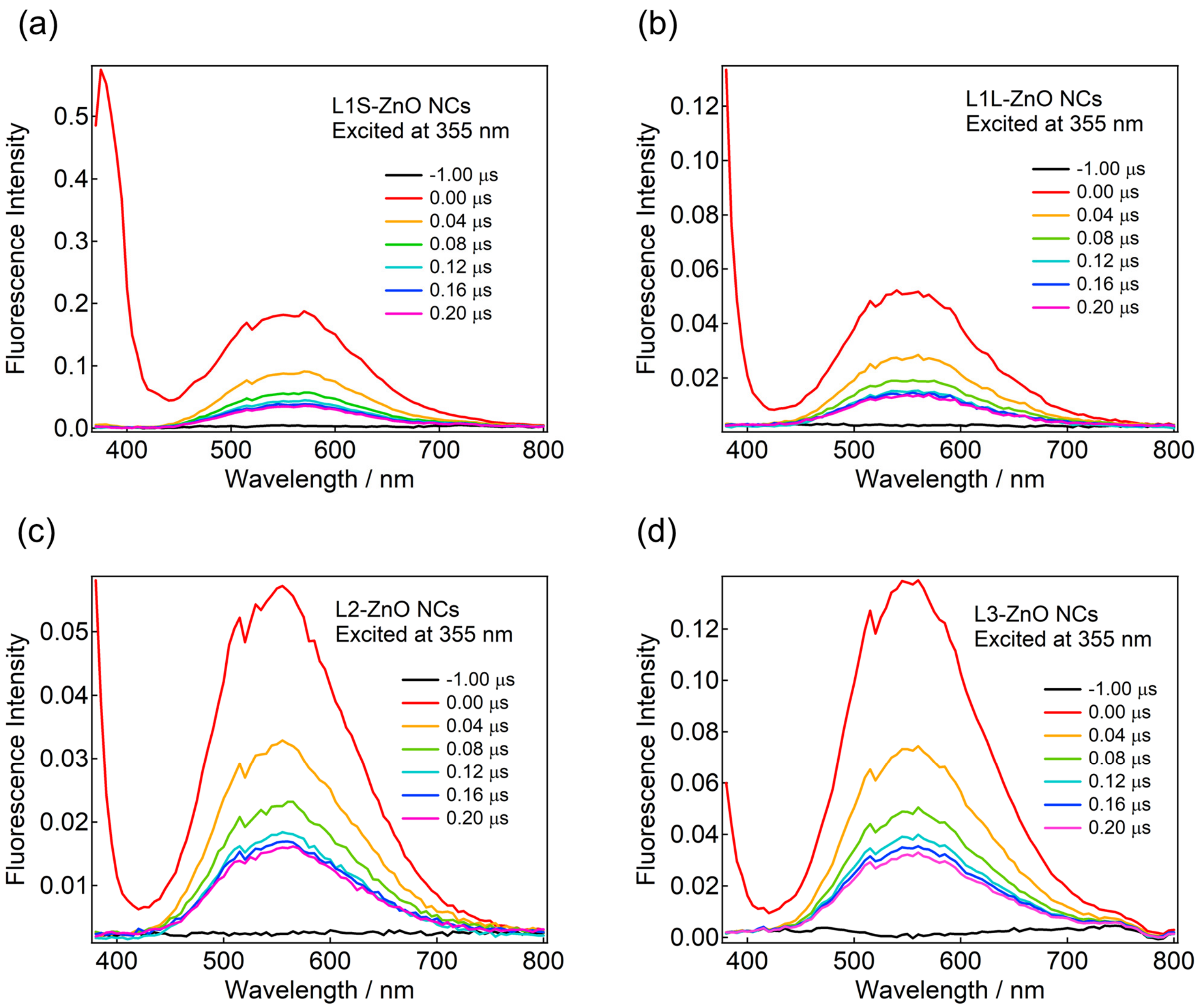

Figure S16. Decay of ZnO NCs Fluorescence spectra

10. Femtosecond-to-Nanosecond Transient Absorption Spectra of ZnO NCs

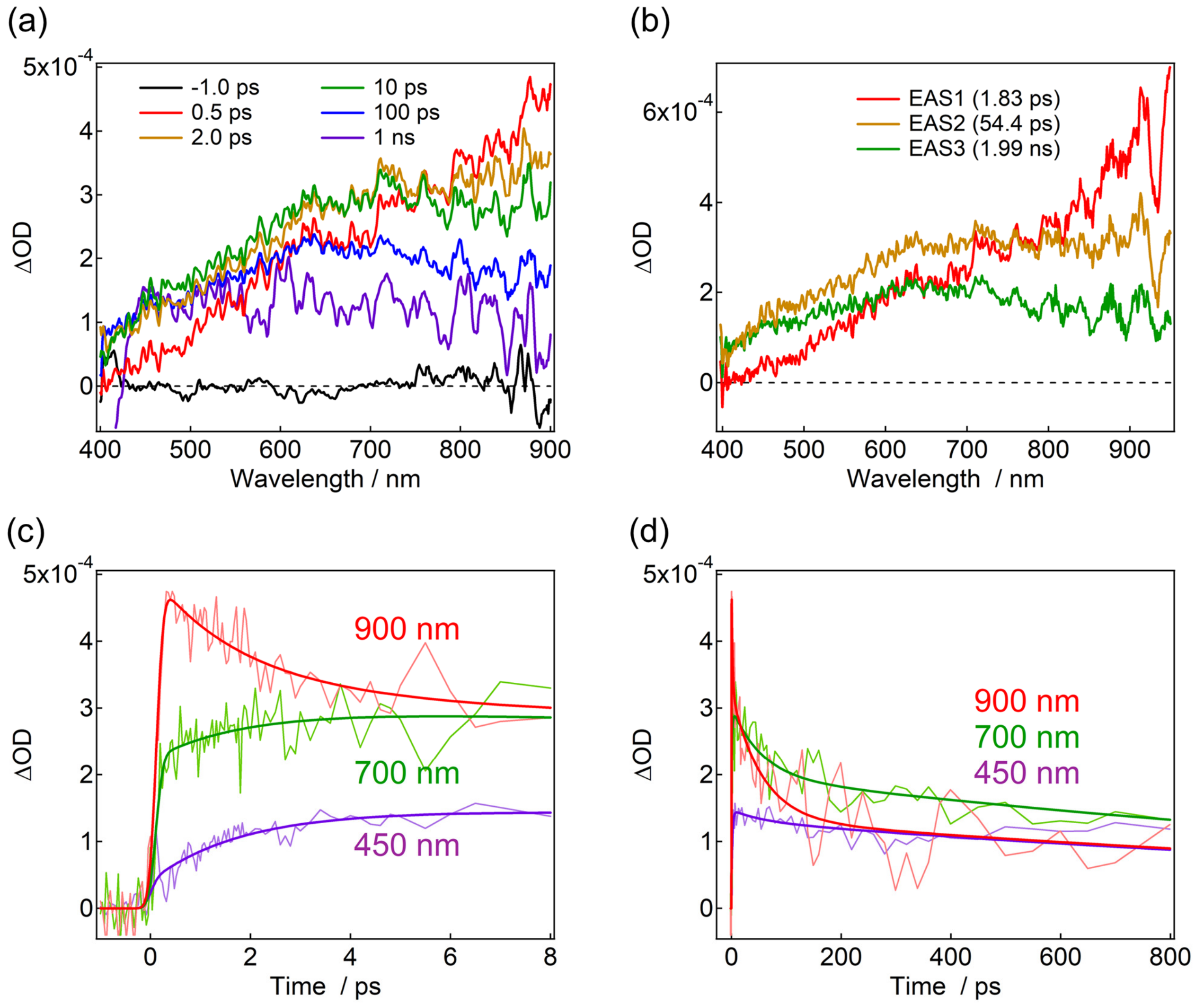

Figure S17. Subpicosecond-to-nanosecond transient absorption (a) spectra, (b) evolution-associated-difference spectra (EADS), (c and d) dynamics of L1S-ZnO NCs excited with a 350-nm femtosecond laser pulse (80 nJ/pulse).

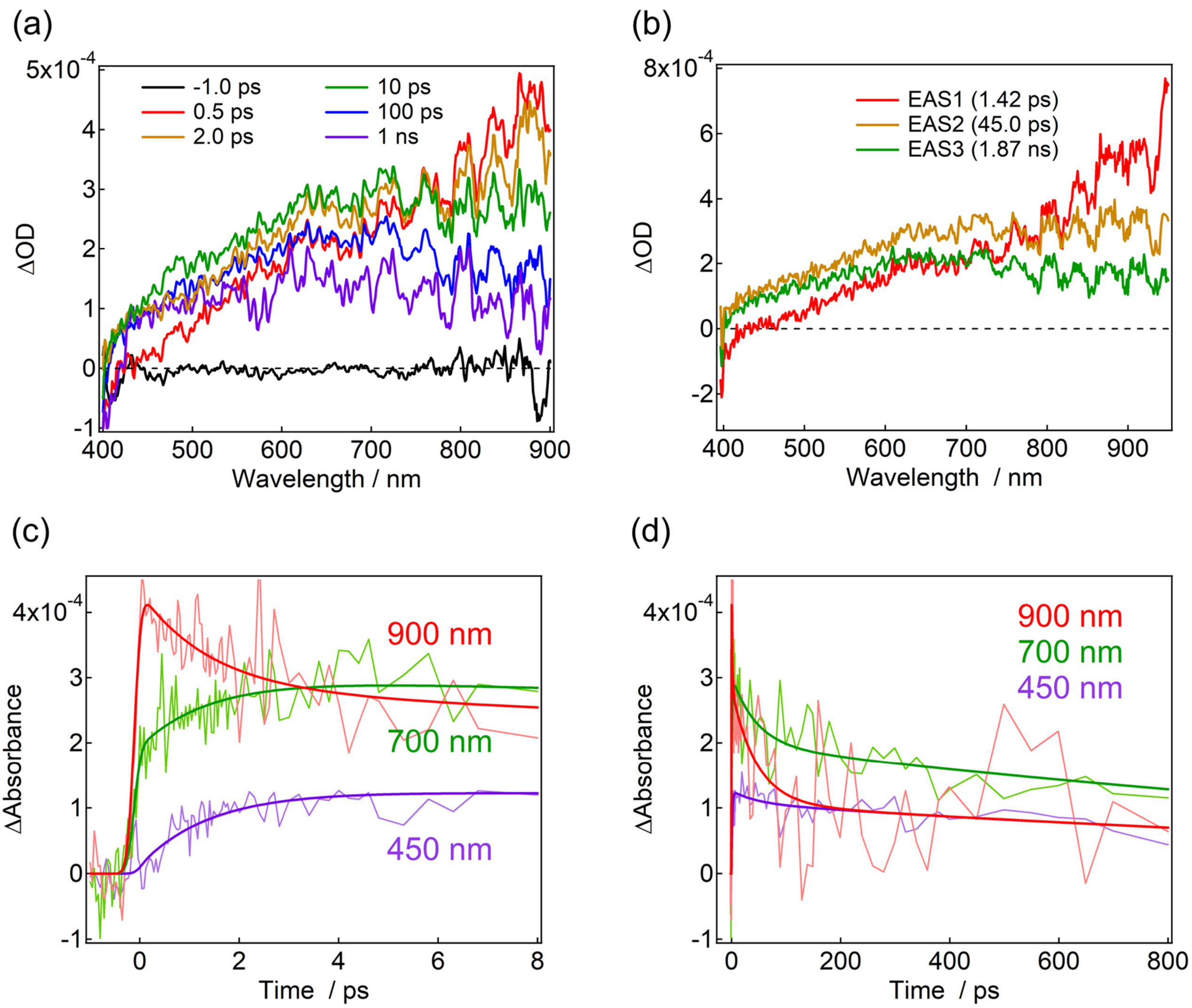

Figure S18. Subpicosecond-to-nanosecond transient absorption (a) spectra, (b) evolution-associated-difference spectra (EADS), (c and d) dynamics of L1L-ZnO NCs excited with a 350-nm femtosecond laser pulse (80 nJ/pulse).

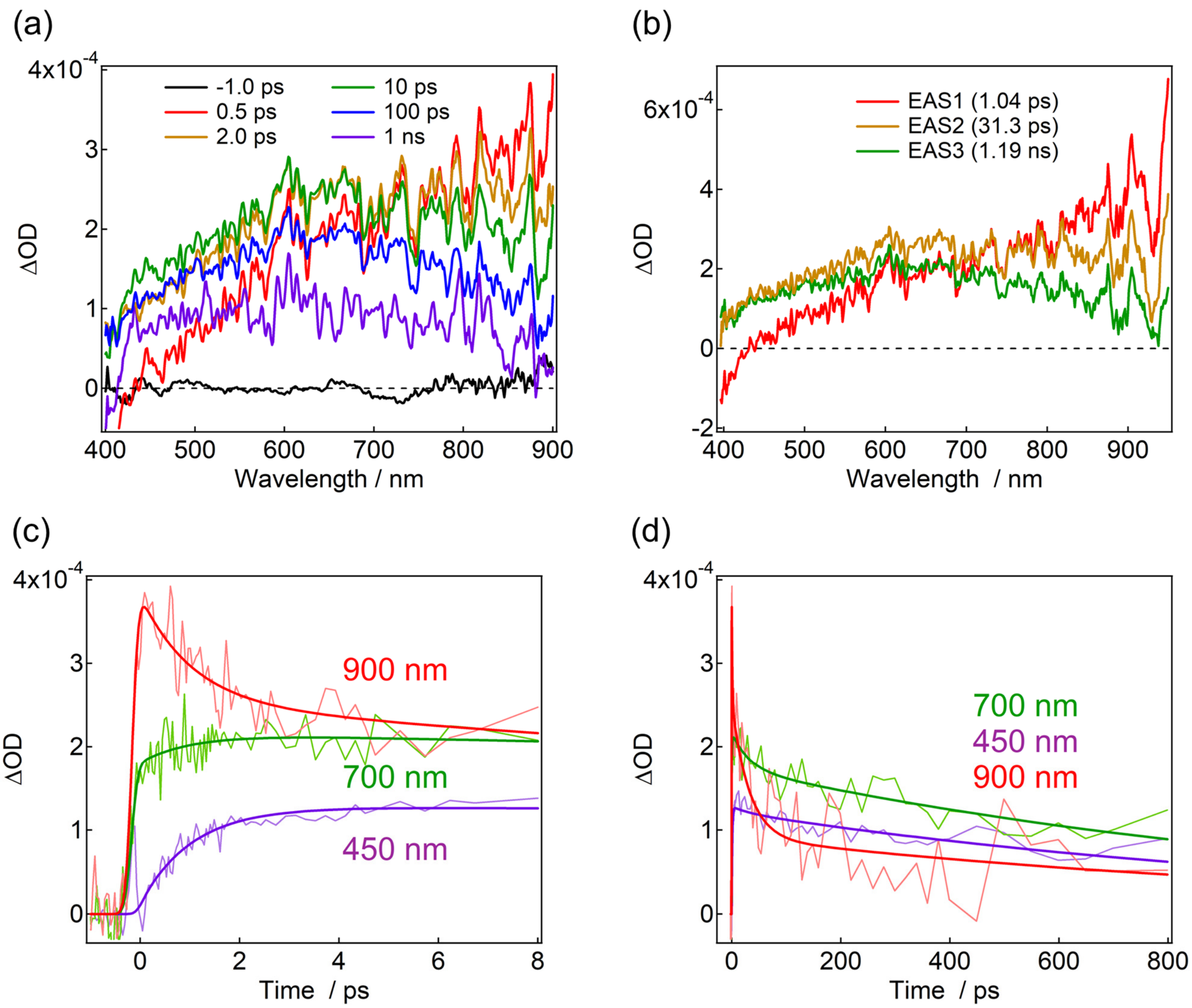

Figure S19. Subpicosecond-to-nanosecond transient absorption (a) spectra, (b) evolution-associated-difference spectra (EADS), (c and d) dynamics of L2-ZnO NCs excited with a 350-nm femtosecond laser pulse (80 nJ/pulse).

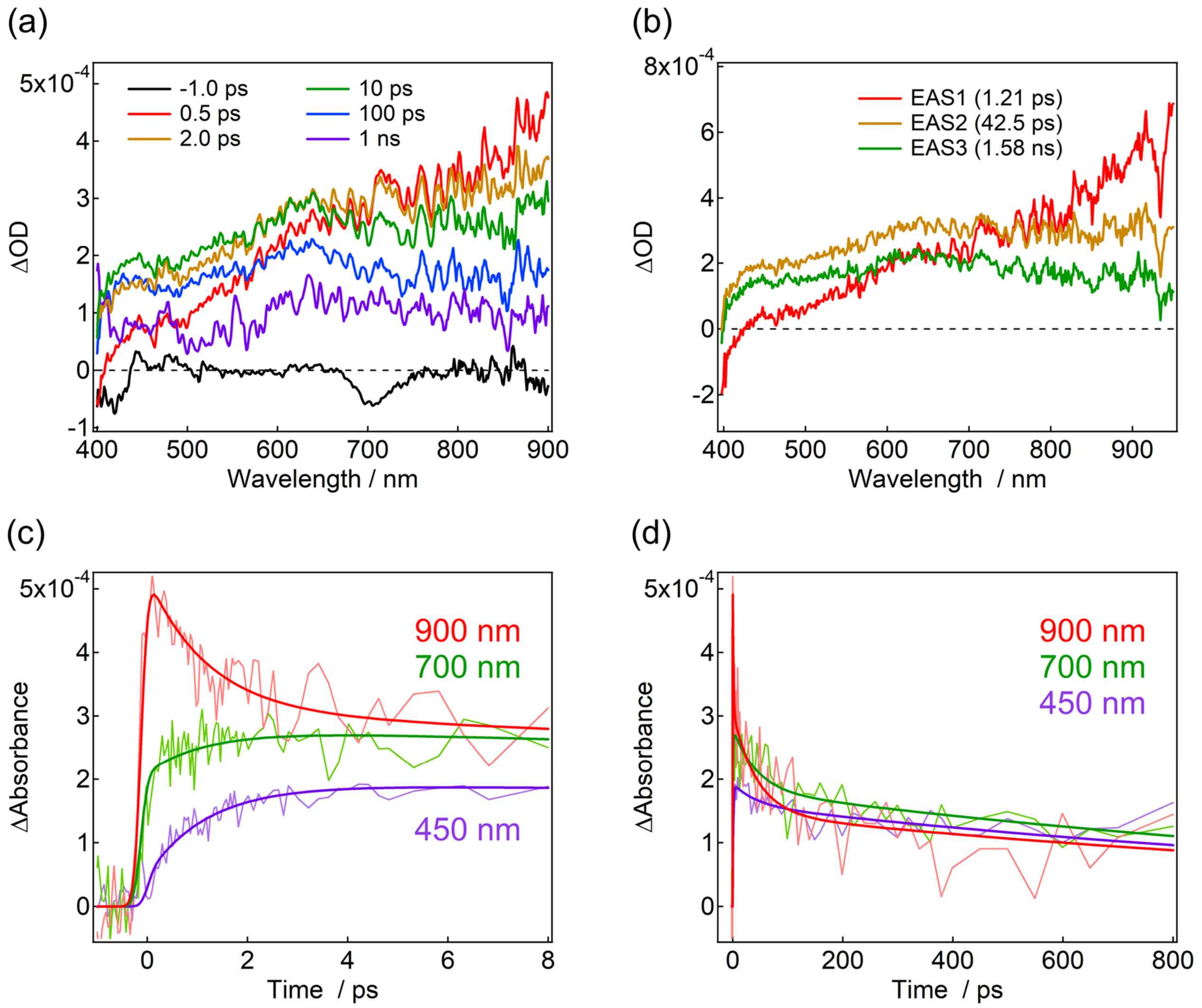

Figure S20. Subpicosecond-to-nanosecond transient absorption (a) spectra, (b) evolution-associated-difference spectra (EADS), (c and d) dynamics of L3-ZnO NCs excited with a 350-nm femtosecond laser pulse (80 nJ/pulse).

Table S2. Standard deviations of time constants caused by global analyses.

	L1S-ZnO NCs	L1L-ZnO NCs	L2-ZnO NCs	L3-ZnO NCs
EAS1	1.83 ± 0.02 ps	1.42 ± 0.02 ps	1.04 ± 0.01 ps	1.21 ± 0.01 ps
EAS2	54.4 ± 0.9 ps	45.0 ± 1.0 ps	31.3 ± 0.5 ps	42.5 ± 0.7 ps
EAS3	1990 ± 50 ps	1870 ± 40 ps	1190 ± 10 ps	1580 ± 30 ps

Table S3. Standard deviations of time constants caused by different datasets.

	L1S-ZnO NCs	L1L-ZnO NCs	L2-ZnO NCs	L3-ZnO NCs
EAS1	2.1 ± 0.3 ps	1.50 ± 0.08 ps	1.08 ± 0.04 ps	1.33 ± 0.12 ps
EAS2	62 ± 8 ps	55 ± 10 ps	35 ± 3 ps	50 ± 7 ps
EAS3	2.4 ± 0.4 ns	2.7 ± 0.8 ns	1.32 ± 0.13 ns	1.3 ± 0.2 ns

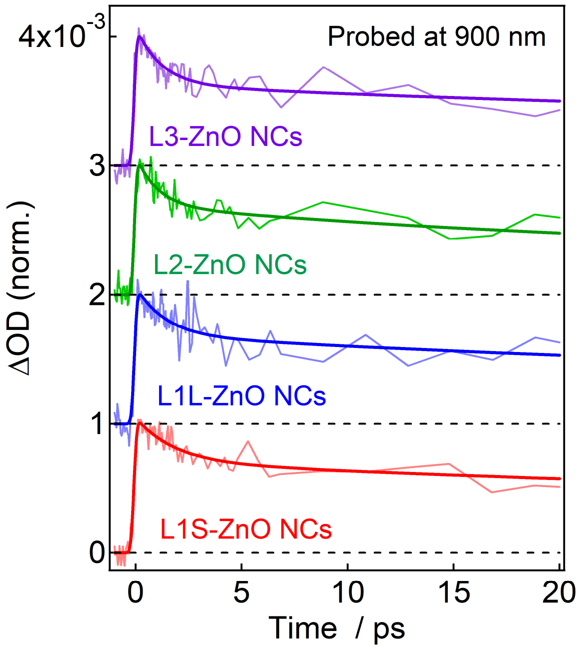

Figure S21. Transient absorption dynamics of ZnO NCs capped with different phosphonic acids excited at 350 nm and probed at 900 nm.

11. References

(1) S. Yamashita, Y. Ito, H. Kamiya and Y. Okada, *Adv. Powder Technol.* **2023**, accepted, APT 104277.

(2) R. Escudero and R. Escamilla. *Solid State Commun.*, 2011, **151**, 97.

(3) N. C. Anderson, M. P. Hendricks, J. J. Choi, and J. S. Owen *J. Am. Chem. Soc.* **2013**, *135*, 18536.
